# Author Correction: The mechanism of low blue light-induced leaf senescence mediated by GmCRY1s in soybean

**DOI:** 10.1038/s41467-024-46224-9

**Published:** 2024-02-26

**Authors:** Zhuang Li, Xiangguang Lyu, Hongyu Li, Qichao Tu, Tao Zhao, Jun Liu, Bin Liu

**Affiliations:** State Key Laboratory of Crop Gene Resources and Breeding, Institute of Crop Sciences, Chinese Academy of Agricultural Sciences, Beijing, China

**Keywords:** Light responses, Plant signalling, Plant molecular biology

Correction to: *Nature Communications* 10.1038/s41467-024-45086-5, published online 27 January 2024

The original version of this article contained an error in Fig. 2h, in which the 3rd, 4th, 5th and 6th lanes of the immunoblot were labelled “3×Flag-GmRGAa/WT”, “3×Flag-GmRGAb/WT”, “3×Flag-GmRGAa/WT”, “3×Flag-GmRGAb/WT” respectively instead of “3×Flag-GmRGAa/ *Gmcry1s-qm*”, “3×Flag-GmRGAb/ *Gmcry1s-qm*”, “3×Flag-GmRGAa/ *GmCRY1b-YFP-1*”, “3×Flag-GmRGAb/ *GmCRY1b-YFP-1*”. This has been corrected in both the PDF and HTML versions of the article.

