# Supplementary material for: The mechanism of low blue light-induced leaf senescence mediated by GmCRY1s in soybean
Source: Nat Commun. 2024 Jan 27;15:798. doi: 10.1038/s41467-024-45086-5 (PMC10821915; doi:10.1038/s41467-024-45086-5)
Supplement: Supplementary file 1 — Supplementary Information [file 41467_2024_45086_MOESM1_ESM.pdf]

Supplementary information

The mechanism of low blue light-induced leaf senescence mediated by GmCRY1s in soybean

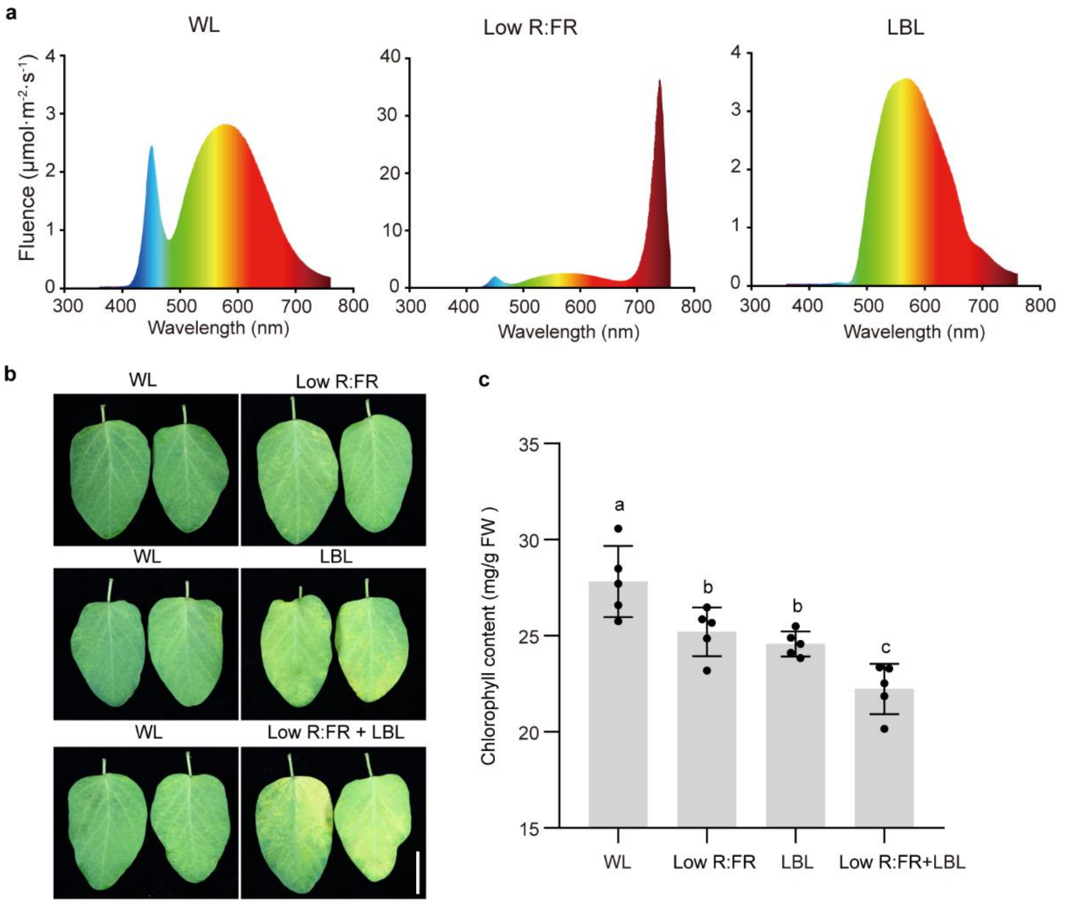

Supplementary Fig. 1: Leaf senescence phenotypic analysis of soybean seedlings under different simulated shade conditions. a Light spectral composition of WL, low R:FR, and LBL. **b** Leaf senescence phenotype of soybean cultivar TL1 under different shade conditions**.** Seedlings were grown under long-day conditions for 10 days, then one of the unifoliate leaves was treated with indicated simulated shade conditions, and another unifoliate leaf was used as a control for 10 days. Low R:FR was achieved by supplementing extra far-red light; LBL was achieved by filtering WL through two layers of yellow filters; Low R:FR+LBL was achieved by supplementing extra far-red light on the basis of LBL. Scale bar, 3 cm. **c** Chlorophyll content of the unifoliate leaves under indicated simulated shade conditions. Values are means ± SD (n = 5 biologically independent plants). The lowercase letters indicate significant differences (P < 0.05, ANOVA with Tukey's post hoc test). Source data are provided as a Source Data file.

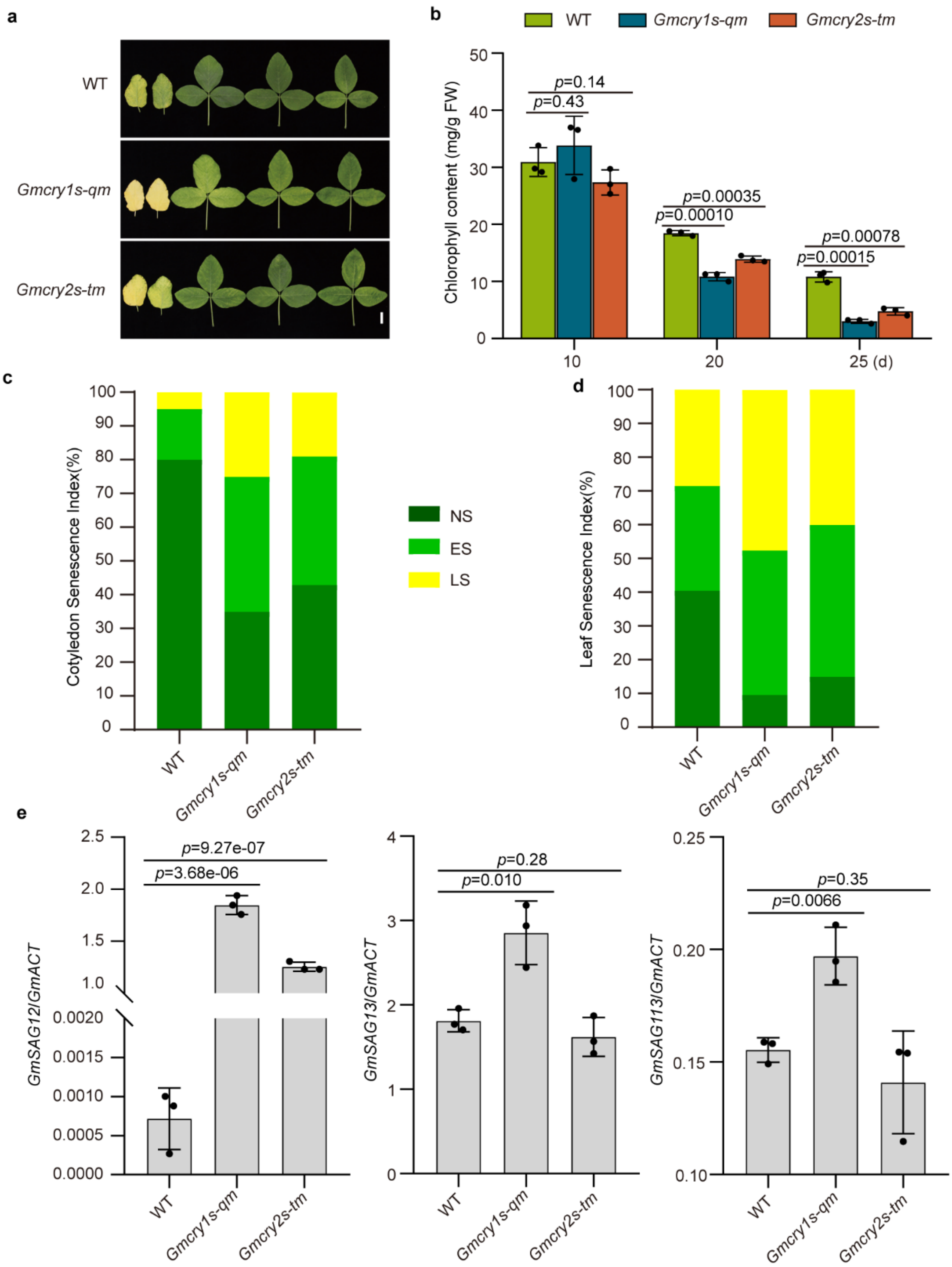

Supplementary Fig. 2: Functional examination of *Gmcrys* in regulating leaf senescence. **a** Leaf senescence phenotype of *Gmcry1s-qm*, *Gmcry2s-tm* mutant, and wild-type plants. Seedlings were grown under long-day (16 h light/8 h dark) conditions for 25 days. Scale bar, 5 cm. **b** Chlorophyll content at the indicated leaf age as in (**a**). Values are means ± SD (n = 3 biological replicates). **c**, **d** Cotyledon senescence index (**c**) and unifoliate leaves senescence index (**d**) as in (**a**). The cotyledons and unifoliate leaf senescence index were calculated at the age of 15 days and 25 days, respectively ($n \geq 10$ biological replicates). **e** Relative transcript levels of senescence marker genes

GmSAG12, *GmSAG13*, and *GmSAG113* at the leaf age of 25 days as in (**a**). Values are means ± SD (n = 3 biological replicates). All above P values were calculated by unpaired two-tailed t-test. Source data are provided as a Source Data file.

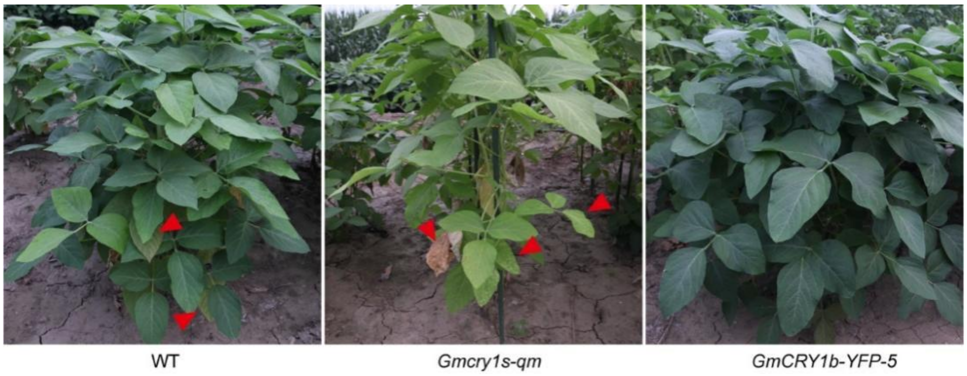

Supplementary Fig. 3: Leaf senescence performance of *Gmcry1s-qm, GmCRY1b-YFP-5*, and wild-type plants under natural field conditions. Representative images of *Gmcry1s-qm* and *GmCRY1b-YFP-5* and wild-type plants grown under natural field conditions at the age of 56 days after sowing. The full red arrows indicate senescent leaves.

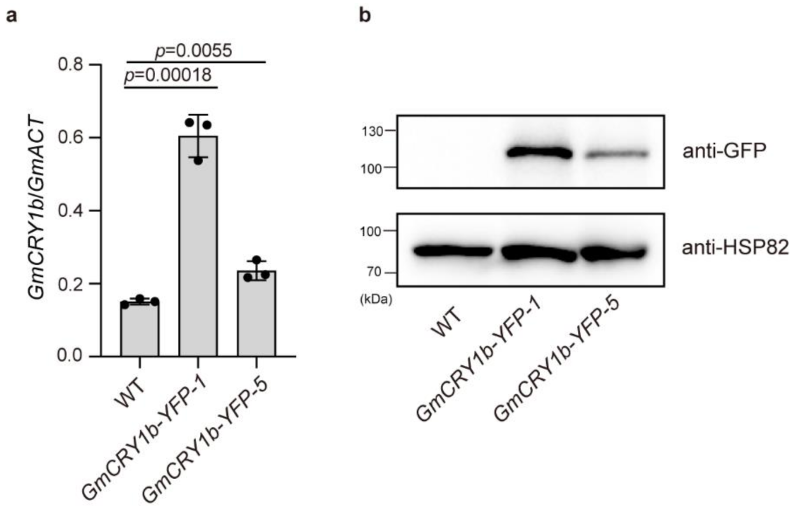

Supplementary Fig. 4: Molecular identification of GmCRY1b overexpression in soybean. a Seedlings were grown under continuous white light for 10 days, then unifoliate leaves were collected for RT-qPCR analysis. *GmActin* was used as an internal control. Values are means ± SD (n = 3 biological replicates). P values were calculated by unpaired two-tailed t-test. **b** Immunoblot analyses show the expression of GmCRY1b-YFP fusion protein in transgenic plants using anti-GFP antibody. The wild-type plants were used as the negative control and HSP82 was used as a loading control. Source data are provided as a Source Data file.

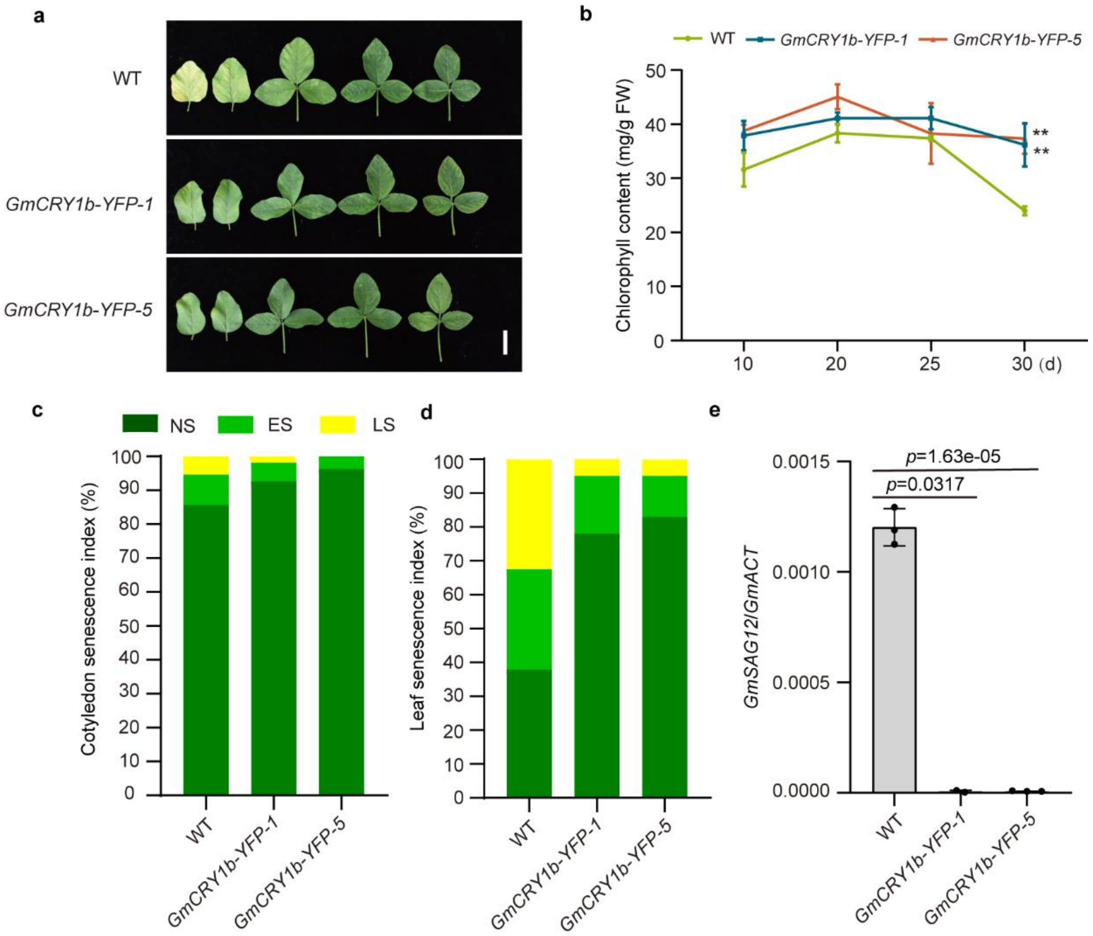

Supplementary Fig. 5: GmCRY1b negatively regulates leaf senescence in soybean. **a** Leaf senescence phenotype of 30-day-old two independent *GmCRY1b* overexpression lines and wild-type plants under long-day (16 h light/8 h dark) conditions. Scale bar, 3 cm. **b** Chlorophyll content in the unifoliate leaf at the indicated leaf age as in (**a**). Values are means ± SD ($n = 5$ biologically independent replicates), $^{**}P < 0.01$. **c**, **d** Cotyledon, and leaf senescence index of two independent *GmCRY1b* overexpression lines and wild-type plants. The cotyledon and unifoliate leaf senescence indexes were calculated at the age of 15 days and 25 days, respectively ($n \geq 20$ biological replicates). **e** Relative transcript levels of senescence marker genes *GmSAG12* in the unifoliate leaves. Seedlings were grown under long-day conditions for 25 days. Values are means ± SD ($n = 3$ biological replicates). P values were calculated by unpaired two-tailed t-test. The *GmActin* gene was used as the internal control. Source data are provided as a Source Data file.

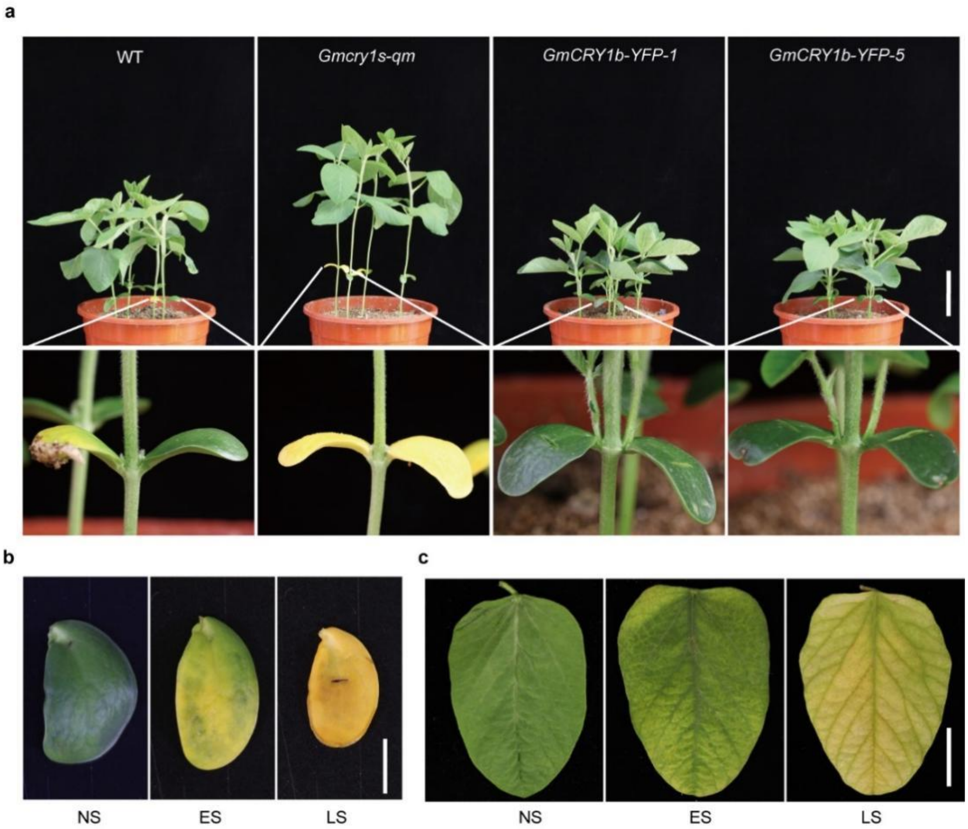

Supplementary Fig. 6: GmCRY1s retard cotyledon senescence in soybean. a Cotyledon senescence phenotypes of wild-type TL1 cultivar, *Gmcry1s-qm*, and two *GmCRY1b* overexpression transgenic seedlings under long-day conditions for 15 days. Scale bar, 5 cm. **b**, **c** Representative cotyledons and leaves images at different stages of senescence. NS, fully expanded mature cotyledons without senescence symptoms; ES, early senescent stage with<25% cotyledons or leaf area yellowing; LS, late senescent stage with over 60% cotyledons or leaf area yellowing. Scale bars, 1 cm in (**b**), and 2 cm in (**c**).

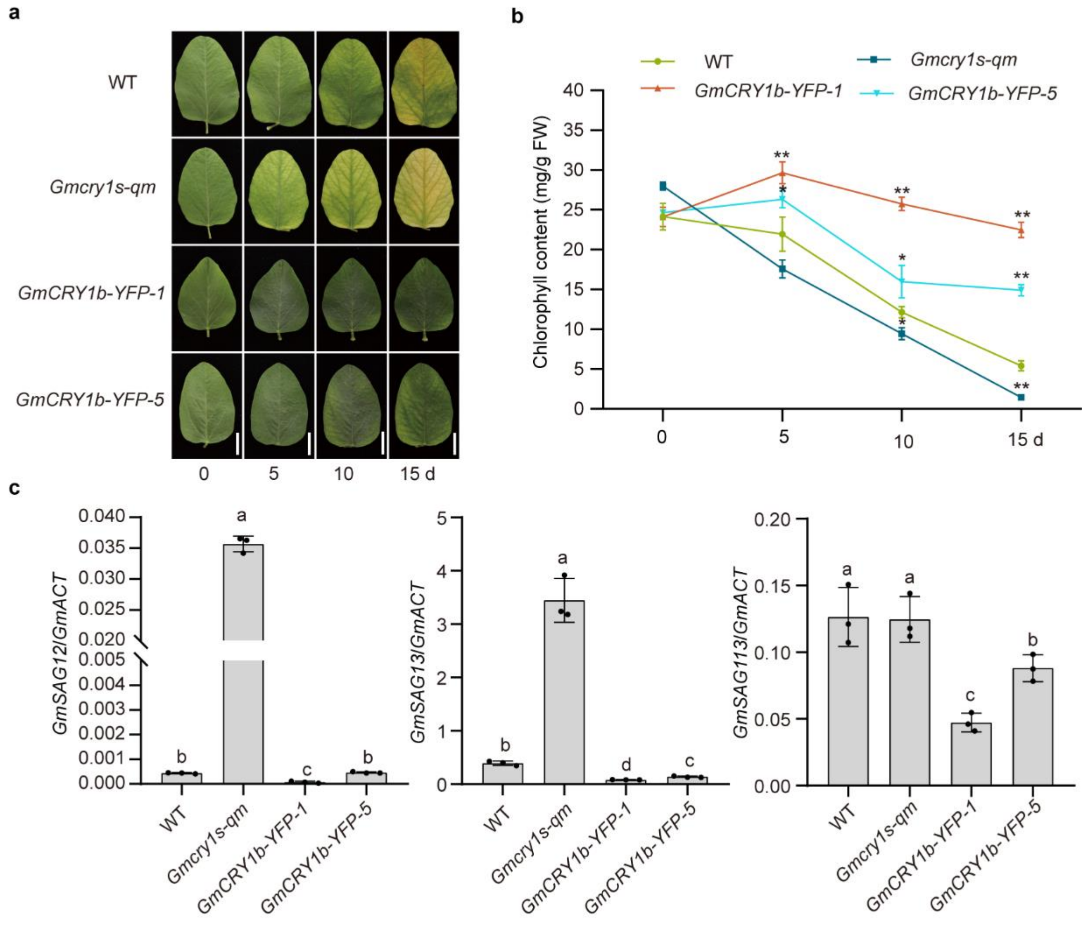

Supplementary Fig. 7: Leaf senescence phenotypic analysis of wild-type plants, *Gmcry1s-qm*, and two independent *GmCRY1b* overexpression lines under detached conditions. a Detached unifoliate leaf senescence phenotype of the indicated lines. Seedlings were grown under continuous light for 10 days, then the unifoliate leaves were detached and incubated in deionized water under continuous light for the indicated days. Scale bars, 2 cm. **b** Chlorophyll content of detached unifoliate leaves under continuous light for the indicated days as in (**a**). Values are means ± SD (n = 3 biological replicates), *P < 0.05, **P < 0.01 by unpaired two-tailed t-test. **c** Relative transcript levels of senescence marker genes *GmSAG12*, *GmSAG13*, and *GmSAG113* under continuous light for 15 days after detachment as in (**a**). Values are means ± SD (n = 3 biological replicates). The lowercase letters indicate significant differences (P < 0.05, ANOVA with Tukey's post hoc test). Source data are provided as a Source Data file.

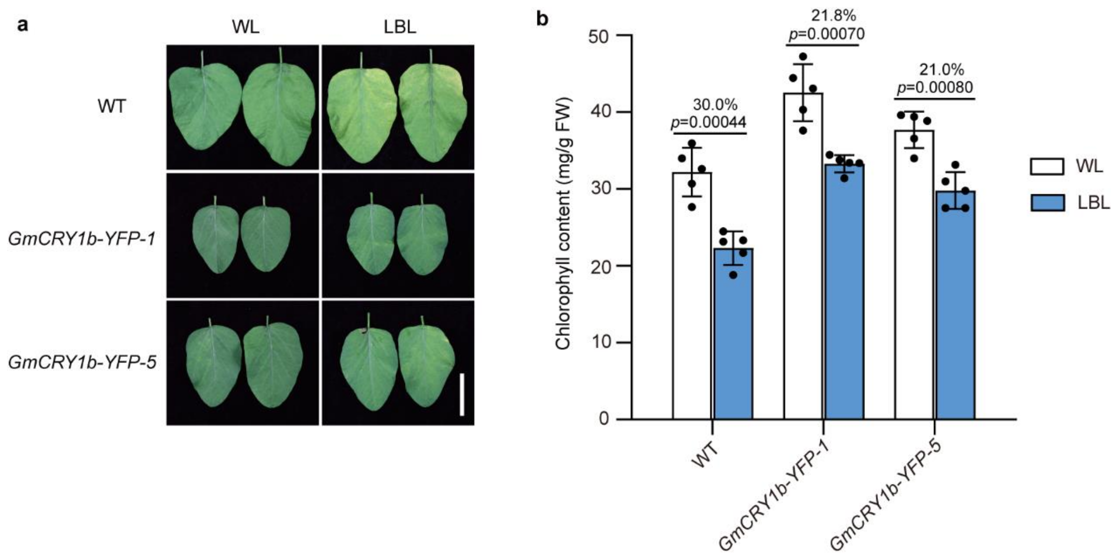

Supplementary Fig. 8: Phenotypic analysis of GmCRY1b in mediating LBL-induced leaf senescence in soybean. a Leaf senescence phenotype of two *GmCRY1b* overexpression lines and wild-type plants under WL or LBL conditions. Seedlings were grown under continuous white light for 10 days, then a pair of unifoliate leaves were treated with different light regimes (LBL or WL) for 10 days. Scale bar, 3 cm. **b** Chlorophyll content in the unifoliate leaves as in (**a**). Values are means ± SD (n = 5 biologically independent plants). P values were calculated by unpaired two-tailed t-test. The percentage decrease in chlorophyll content under WL compared to LBL is indicated by the values above the respective p values. Source data are provided as a Source Data file.

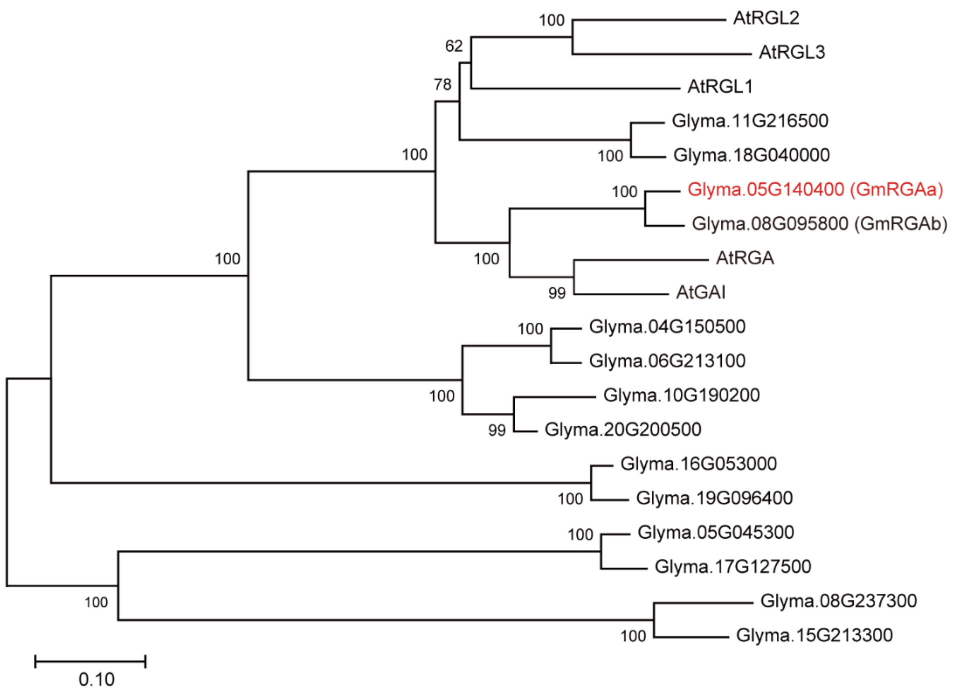

Supplementary Fig. 9: Phylogenetic tree of DELLA proteins in *Arabidopsis* and *Glycine max*. A phylogenetic tree based on the amino acid sequences of DELLA proteins and their homologous proteins in *Arabidopsis* and *Glycine max*. The tree was constructed using the neighbor-joining method. Database for searching: Phytozome (http://phytozome-next.jgj.doe.gov), TAIR10 (http://arabidopsis.org/index.jsp). Highlighted in red in the phylogenetic tree indicates the DELLA protein identified in our yeast two-hybrid (Y2H) screen.

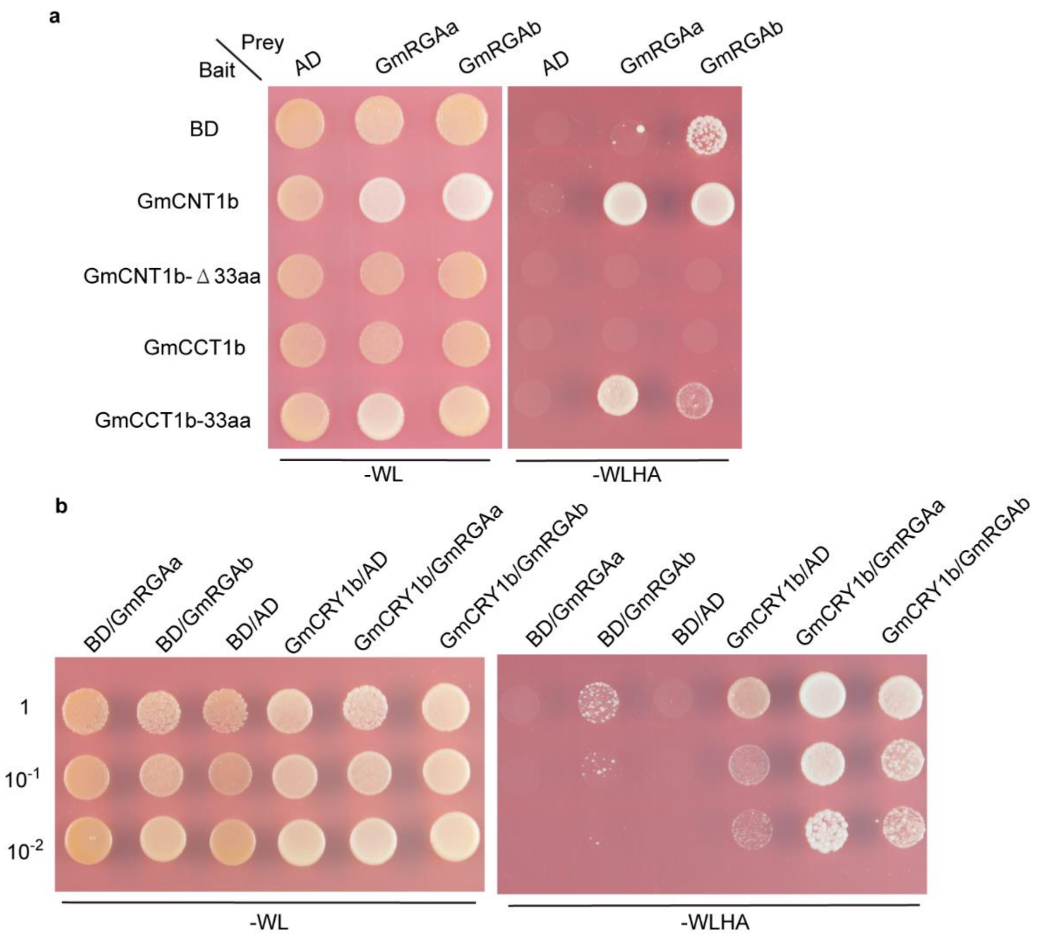

Supplementary Fig. 10: Y2H assays show the interaction of GmCRY1b with DELLA proteins. a The interaction between DELLA proteins and different truncated versions of GmCRY1b. **b** The severe self-activation of GmCRY1b in yeast system. Bait and prey constructs were co-transformed into yeast cells (AH109 strain). The growth of the transformed cells was test in minimal medium lacking Trp and Leu (-WL) and in selective medium lacking Trp, Leu, His, and adenine (-WLHA). Source data are provided as a Source Data file.

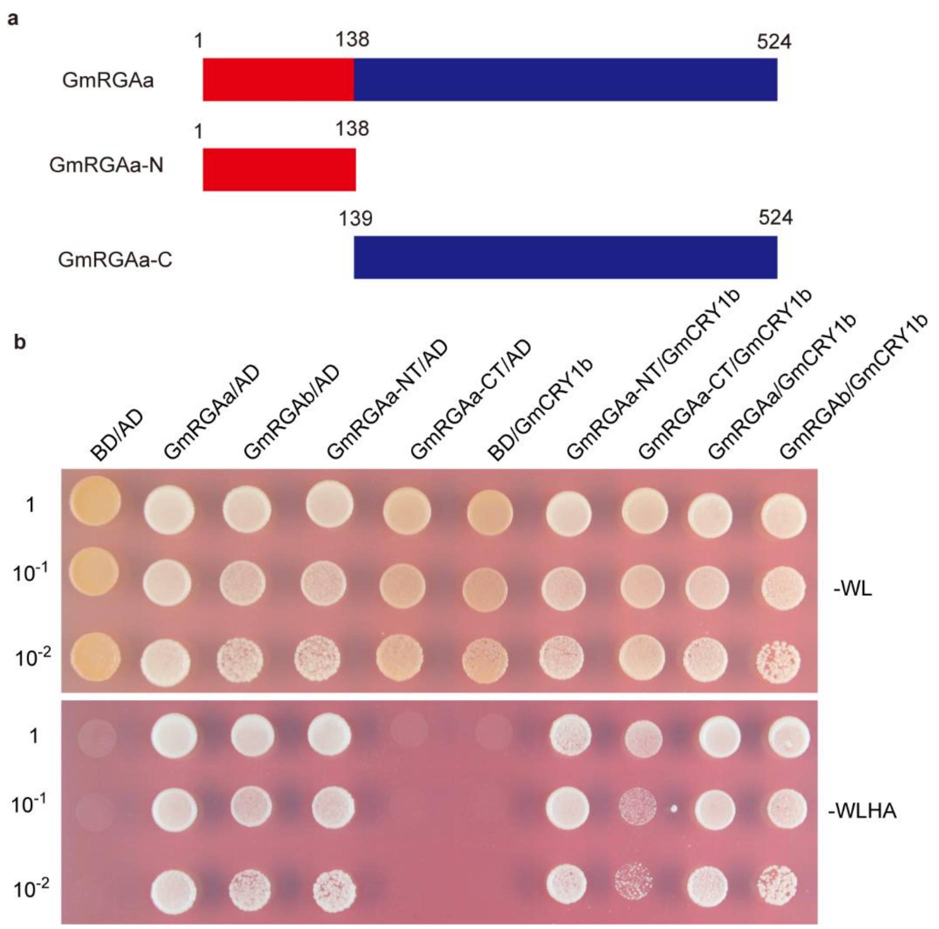

Supplementary Fig. 11: Y2H assays show the autoactivation activity of DELLA proteins GmRGAa and GmRGAb. a Schemes display full-length and truncated versions of DELLA protein GmRGAa. GmRGAa-N, amino-terminal domain of GmRGAa; GmRGAa-C, carboxyl-terminal domain of GmRGAa. **b** The growth of the transformed cells was tested in the indicated medium. Source data are provided as a Source Data file.

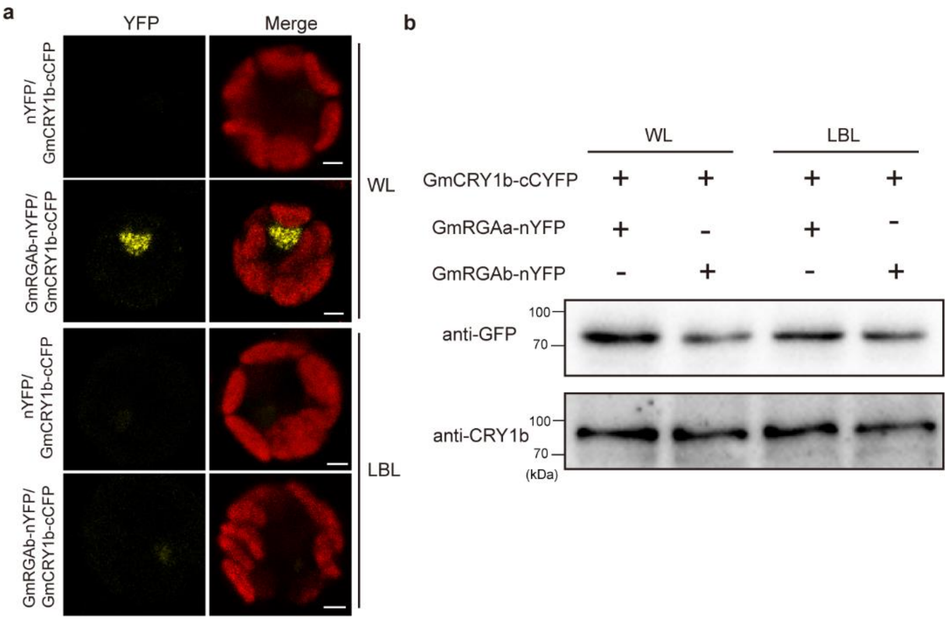

Supplementary Fig. 12: The interaction between GmCRY1b and GmRGAb under LBL conditions. a BIFC assays show the interaction between GmCRY1b and GmRGAb under LBL conditions in soybean mesophyll protoplasts. Scale bars, 2 μM. **b** Immunoblot analysis shows the expression of GmCRY1b, GmRGAa, and GmRGAb proteins in soybean mesophyll protoplasts under WL or LBL conditions. GmCRY1b-cCFP proteins were detected by the anti-GmCRY1b antibody. GmRGAa-nYFP and GmRGAb-nYFP proteins were detected by the GFP-antibody which recognizes the n-terminal region of GFP protein. Source data are provided as a Source Data file.

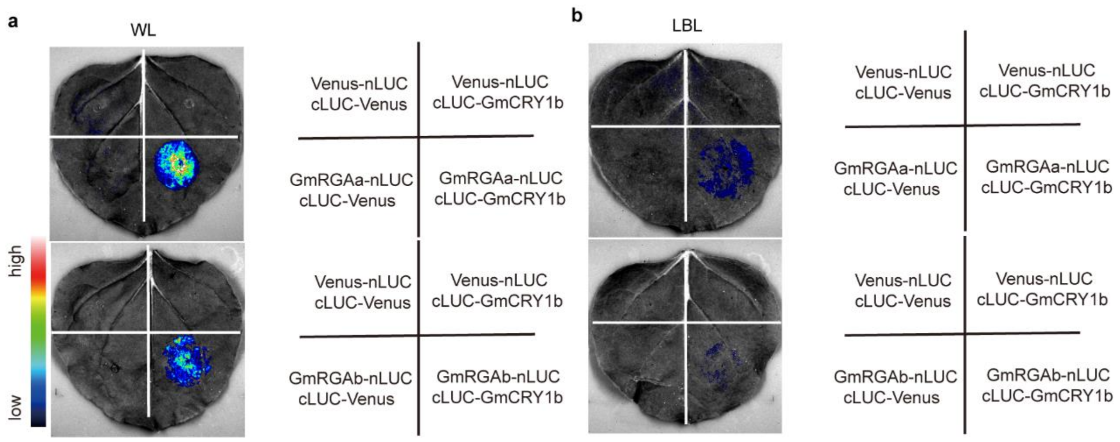

Supplementary Fig. 13: LBL disrupts the interaction between GmCRY1b and DELLA proteins GmRGAa and GmRGAb. The Split-luciferase complementation imaging assays show the interaction of GmCRY1 with DELLA proteins GmRGAa and GmRGAb under WL (**a**) and LBL (**b**) conditions, respectively.

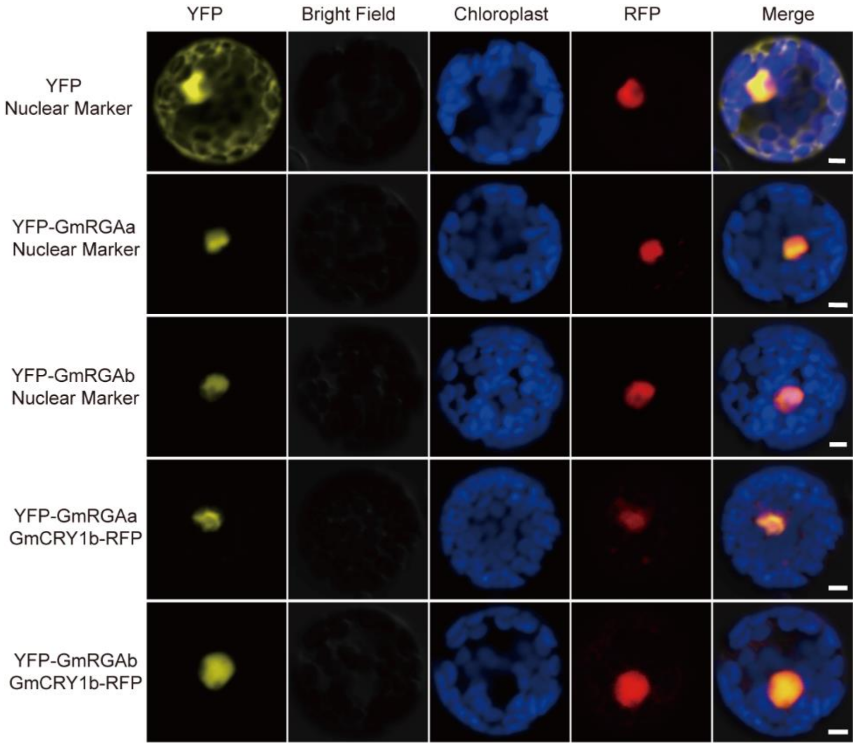

Supplementary Fig. 14: Subcellular localization of GmCRY1b and DELLA proteins GmRGAa and GmRGAb in *Arabidopsis* protoplasts. GmMYB29 fused with RFP was used as a nuclear marker. YFP-GmRGAa and YFP-GmRGAb constructs were transiently co-transformed with GmMYB29-RFP construct in *Arabidopsis* protoplasts, respectively. An empty vector (YFP only) was used as a control. Scale bars, 5 μM.

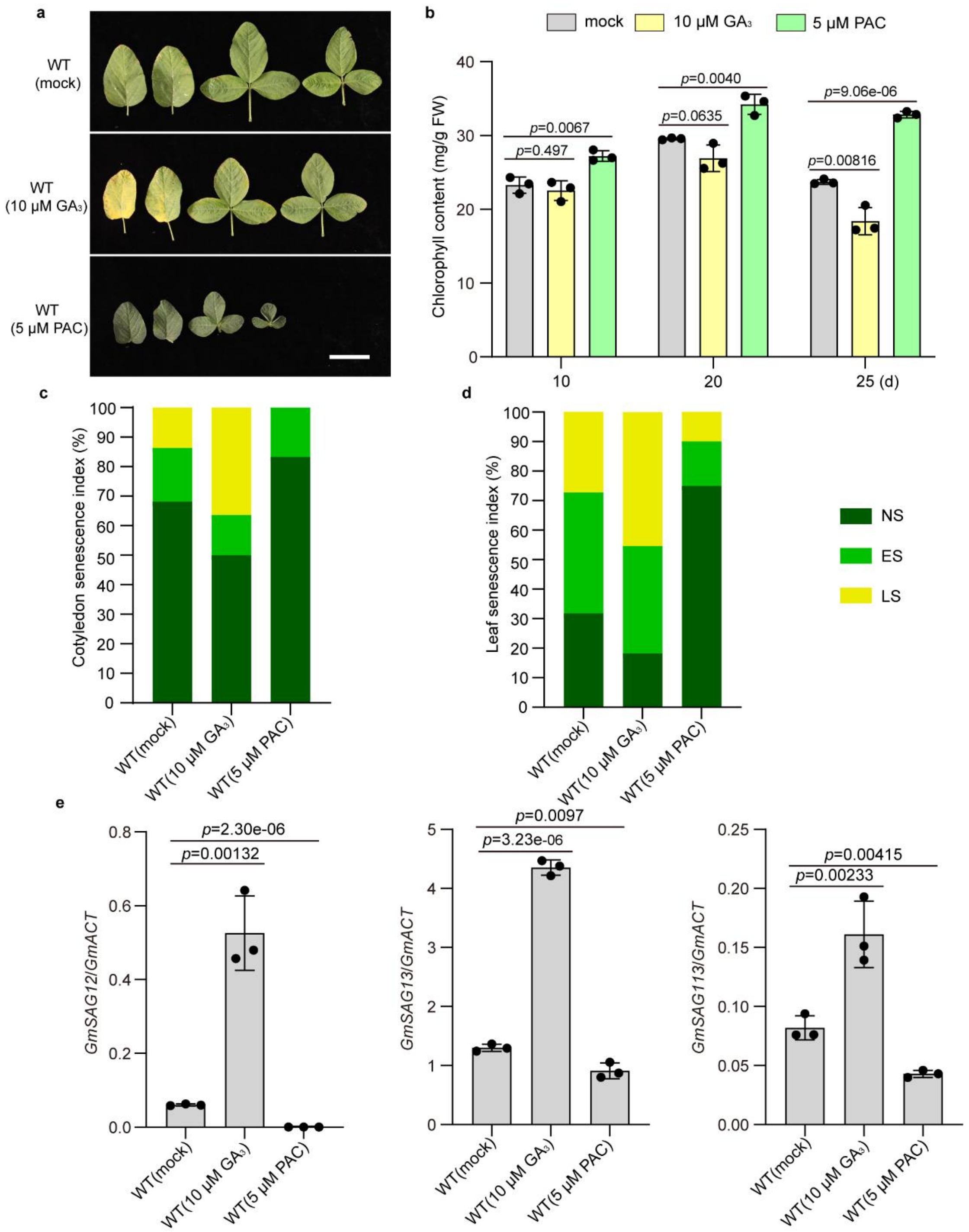

Supplementary Fig. 15: Analysis of the effect of gibberellin on the progress of leaf senescence in soybean. a leaf senescence phenotype of wild-type TL1 cultivar treated with 10 µM GA_3 and 5 µM PAC under long-day conditions for 25 days. Seedlings were de-etiolated for 6 days, then exogenously treated with 10 µM GA_3 and 5 µM PAC for 19 days. Scale bar, 5 cm. **b** Chlorophyll content in the unifoliate leaf at the indicated leaf age as in (**a**). Values are means ± SD (n = 3 biological replicates). **c, d** Cotyledon senescence index (**c**) and unifoliate leaf senescence index (**d**) as in (**a**), which were calculated at the age of 15 days and 25 days, respectively ($n \geq 20$ biological replicates).

e Relative transcript levels of senescence marker genes *GmSAG12*, *GmSAG13,* and *GmSAG113* in the unifoliate leaf at the leaf age of 25 days as in (**a**). Values are mean ± SD (n = 3 biological replicates). All above P values were calculated by unpaired two-tailed t-test. Source data are provided as a Source Data file.

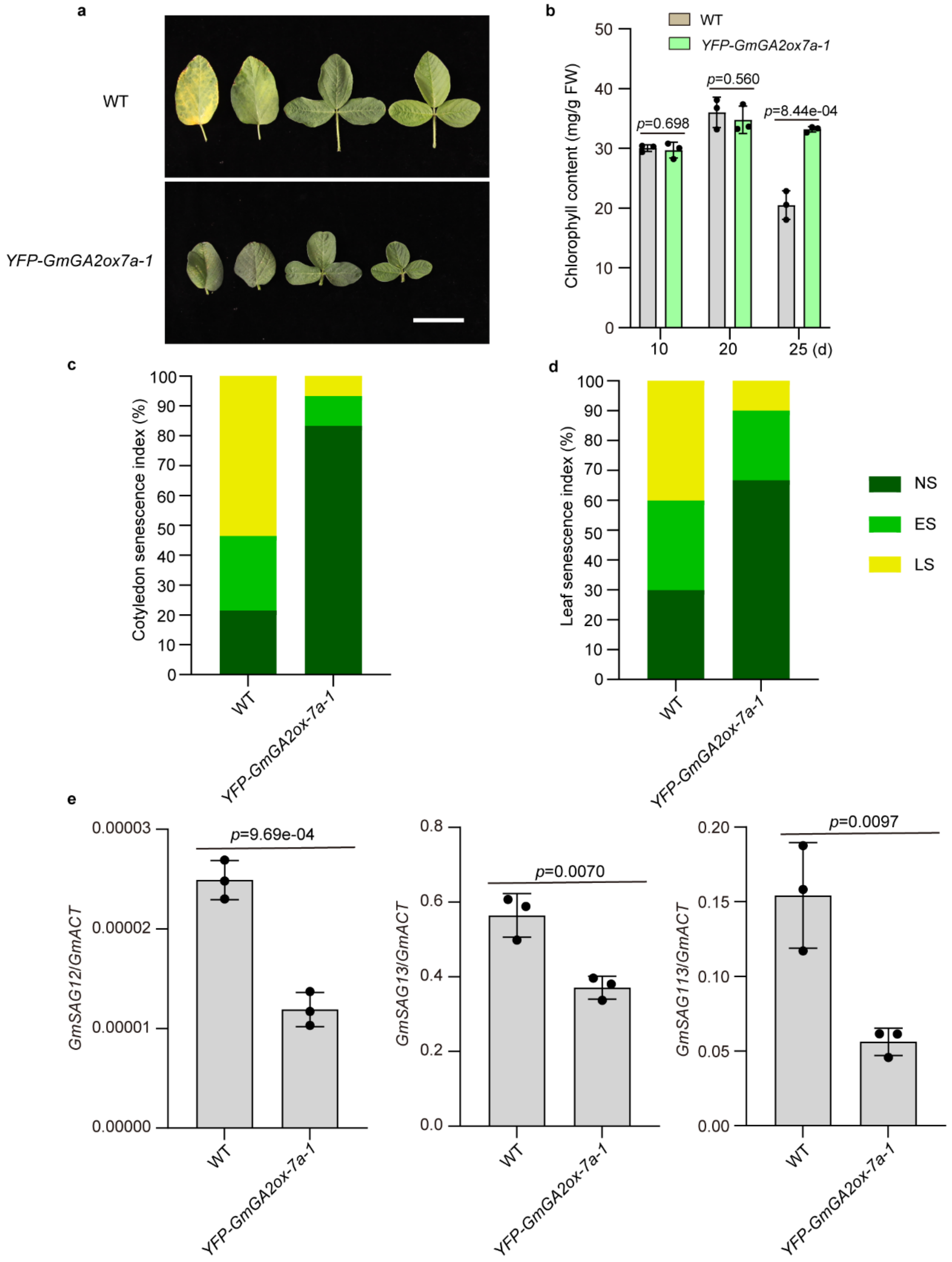

Supplementary Fig. 16: Comparison of the leaf senescence phenotype between the wild-type plants and the *YFP-GmGA2ox-7a* overexpression line. a Leaf images of indicated plants grown under long-day conditions for 25 days. Scale bar, 5 cm. **b**

Chlorophyll content in the unifoliate leaf at the indicated leaf age as in (**a**). Values are means ± SD (n = 3 biological replicates). **c, d** Cotyledon senescence index (**c**) and unifoliate leaf senescence index (**d**) as in (**a**), which were calculated at the age of 15 days and 25 days, respectively ($n \geq 10$ biological replicates). **e** Relative transcript levels of senescence marker genes *GmSAG12*, *GmSAG13,* and *GmSAG113* in the unifoliate leaves as in (**a**). Values are mean ± SD (n = 3 biological replicates). All above P values were calculated by unpaired two-tailed t-test. Source data are provided as a Source Data file.

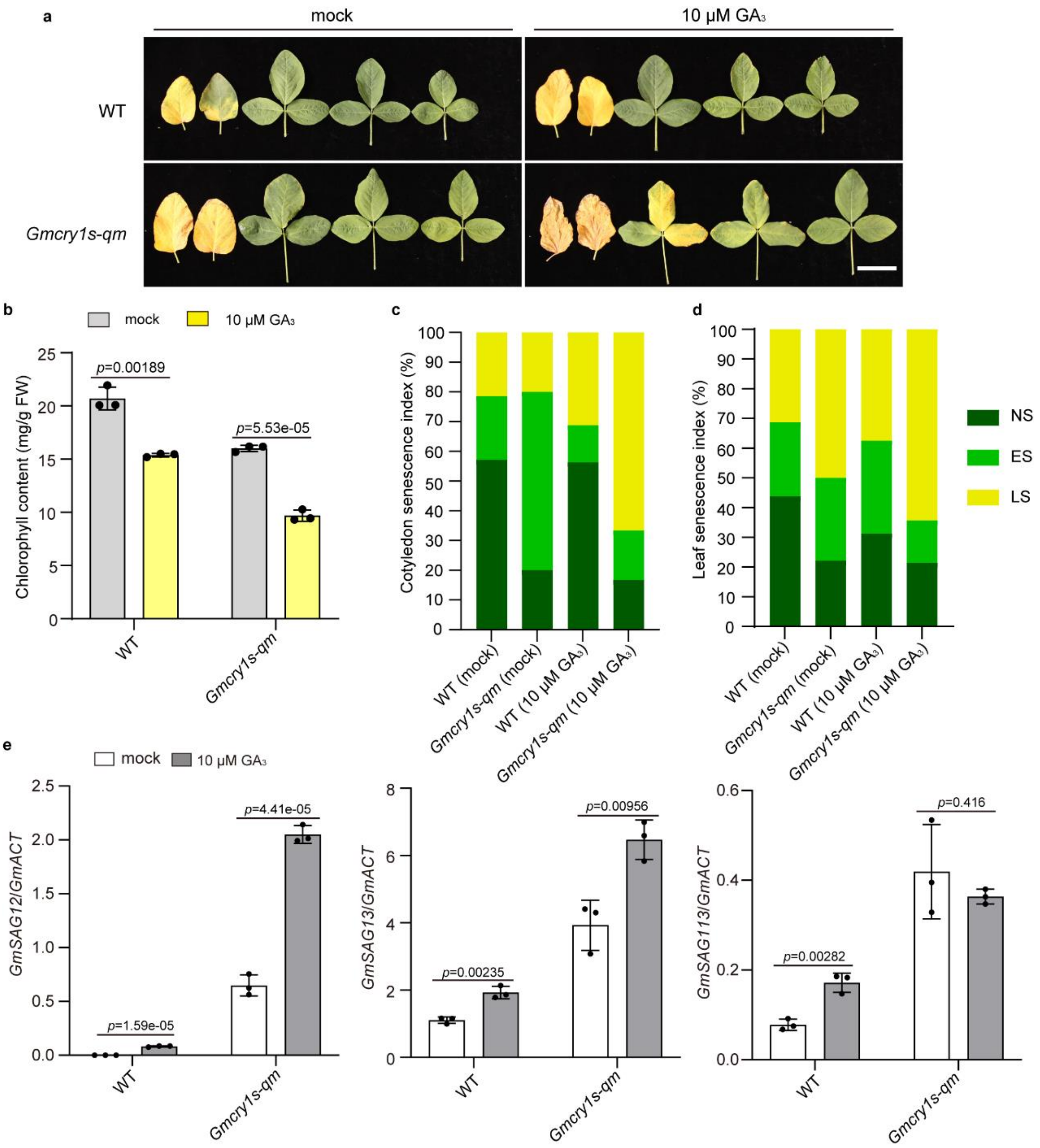

Supplementary Fig. 17: GmCRY1s participate in GA-induced leaf senescence in soybean. a leaf senescence phenotype of wild-type plants and *Gmcry1s-qm* treated with 10 µM GA_3 under long-day conditions for 25 days. Seedlings were de-etiolated for 6 days, then exogenously treated with 10 µM GA_3 for 19 days. Scale bar, 5 cm. **b** Chlorophyll content in the unifoliate leaf at the leaf age of 21 days as in (**a**). Values are means ± SD (n = 3 biological replicates). **c, d** Cotyledon senescence index (**c**) and unifoliate leaf senescence index (**d**) as in (**a**). The cotyledon and unifoliate leaf senescence index was calculated at the age of 15 days and 25 days, respectively ($n \geq 10$ biologically independent plants). **e** Relative transcript levels of senescence marker genes *GmSAG12*, *GmSAG13,* and *GmSAG113* in the unifoliate leaves at the leaf age of 21 days as in (**a**). Values are mean ± SD (n = 3 biological replicates). All above P values

were calculated by unpaired two-tailed *t*-test. Source data are provided as a Source Data file.

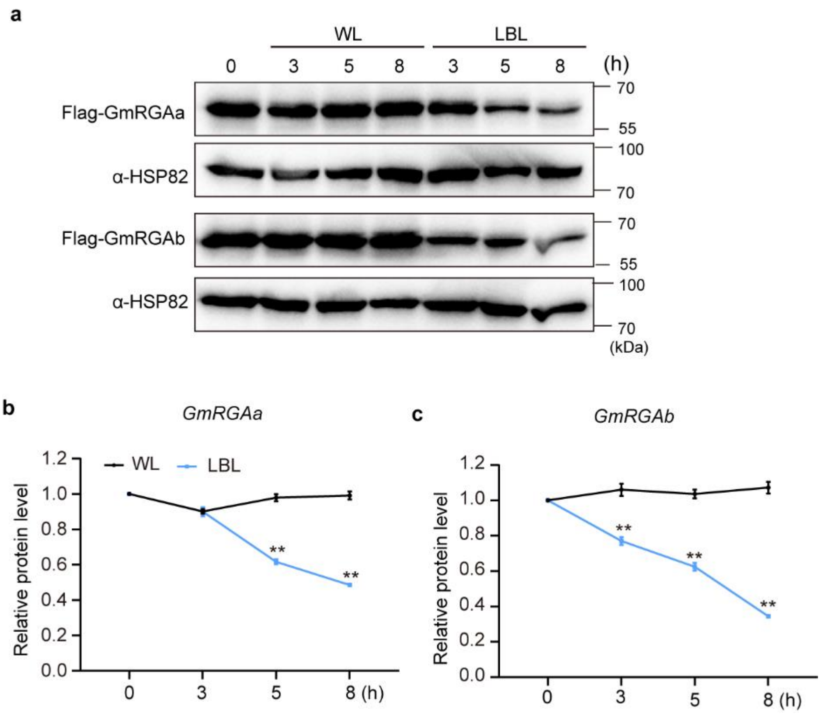

Supplementary Fig. 18: The effect of LBL on the protein stability of DELLA protein GmRGAa and GmRGAb in soybean calluses. a Immunoblot analysis of the protein levels of GmRGAa and GmRGAb in response to LBL treatment. Calluses were cultured under continuous light for 14 days, then were transformed into LBL conditions or kept in the continuous white light for the indicated time course. **b, c** Relative protein levels of Flag-GmRGAa (**b**) and Flag-GmRGAb (**c**) normalized to HSP82 as in (**a**). Values are means ± SD (n = 3 biological replicates), $^{**}P < 0.01$ by unpaired two-tailed *t*-test. Source data are provided as a Source Data file.

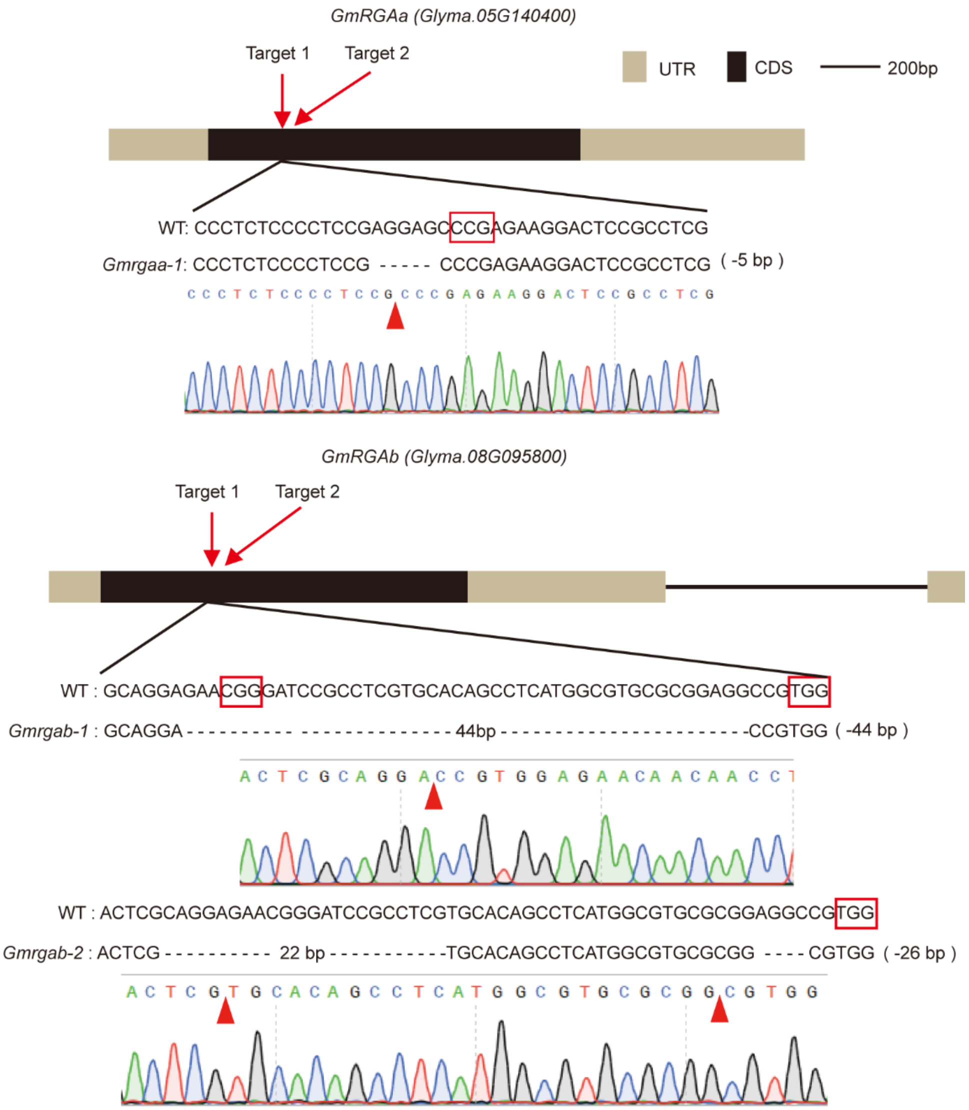

Supplementary Fig. 19: Generation of *Gmrgaa* and *Gmrgab* mutants by CRISPR/Cas9 system in soybean. The long red arrow indicates the target sites of the CRISPR/Cas9 system in the region of the *GmRGAa* and *GmRGAb* genes. The red boxes indicate the protospacer-adjacent motif (PAM). CRISPR/Cas9-induced 5 bp deletion at target 1 site in the exon of *GmRGAa* gene; 44 bp and 22 bp/4 bp deletion at target 1 and target 2 in the exon of *GmRGAb* gene detected by Sanger sequencing, respectively. Red arrowheads represent the cleavage site.

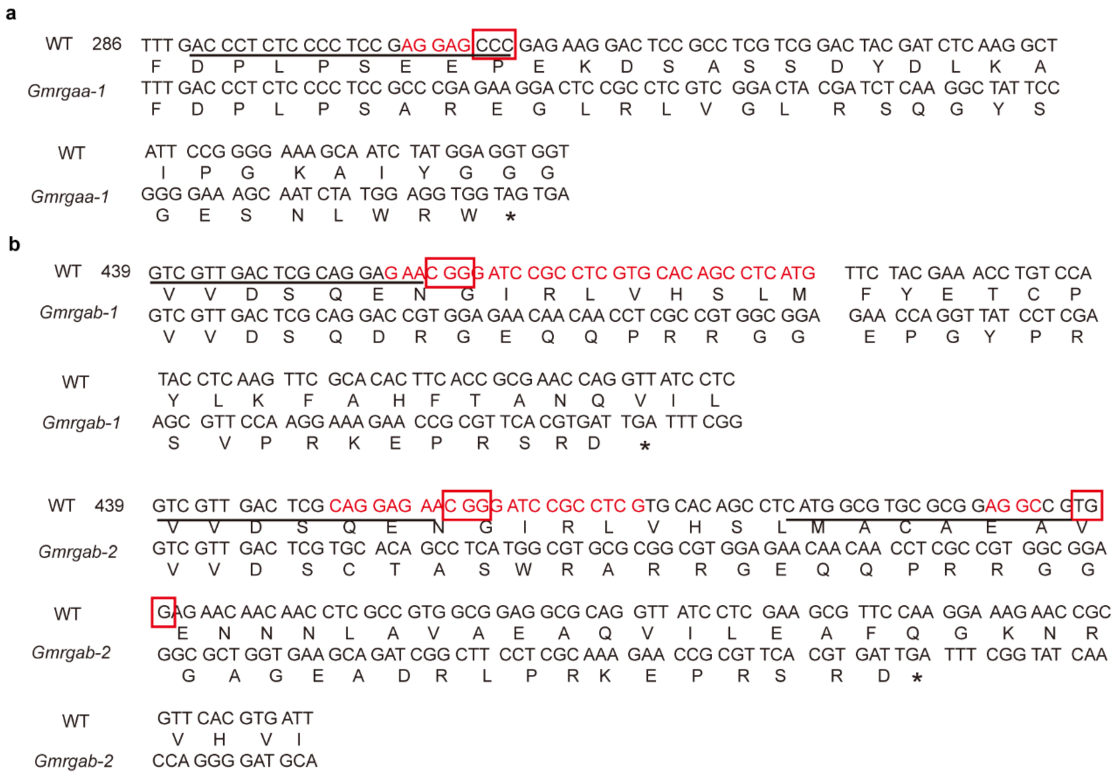

Supplementary Fig. 20: DNA sequence and amino acid sequence comparison of WT and DELLA proteins GmRGAa (a) and GmRGAb (b) mutants. Highlighted in red in the DNA sequence indicate the mutated regions in DELLA proteins *GmRGAa* (**a**) and *GmRGAb* (**b**) mutants. The underlined nucleotides indicate the target sites, and the red boxes indicate the PAM. An asterisk indicates the termination of translation.

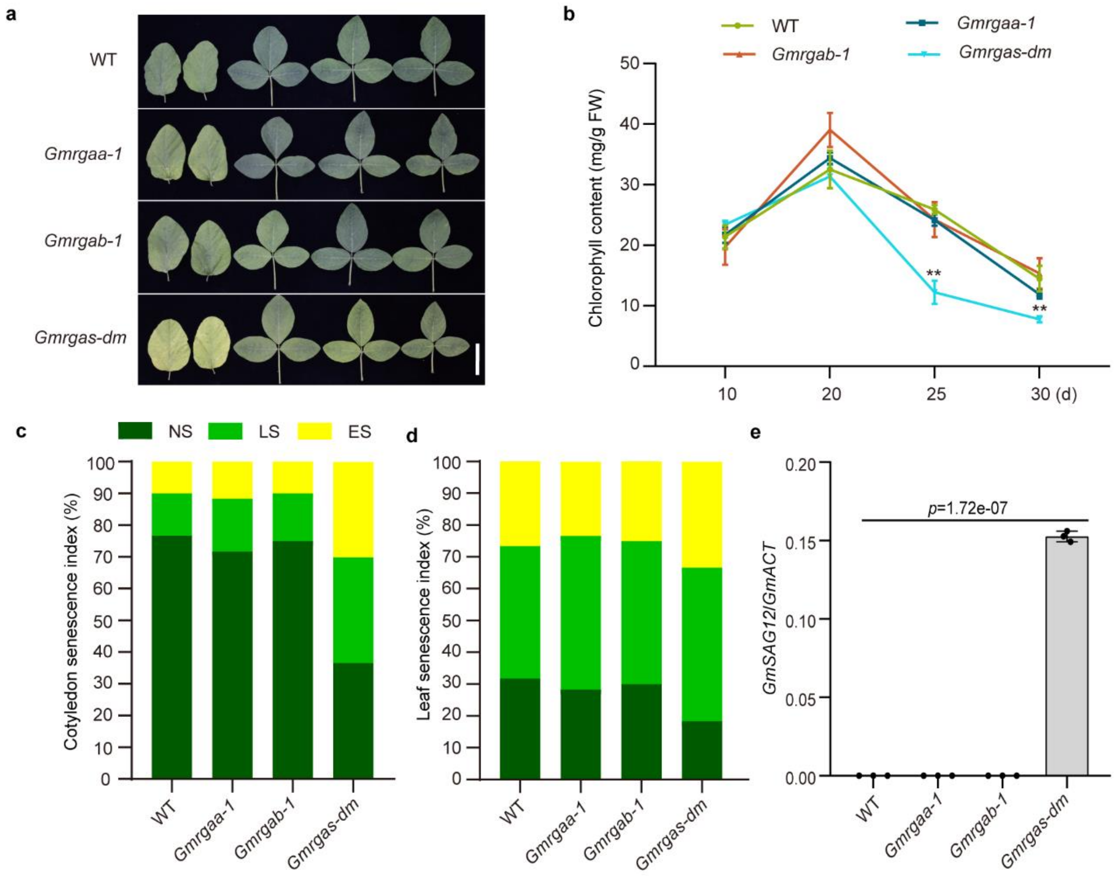

Supplementary Fig. 21: Phenotypic analysis of LBL-induced leaf senescence in the wild-type plants and DELLA protein mutants. a Leaf senescence phenotype of *Gmrgaa-1*, *Gmrgab-1*, *Gmrgas-dm* mutant, and wild-type plants under long-day conditions for 25 days. Scale bar, 5 cm. **b** Chlorophyll content in the leaves at the indicated leaf age as in (**a**). Values are means ± SD (n = 5 biologically independent plants), **$p < 0.01$. **c**, **d** Cotyledon senescence index (**c**) and unifoliate leaf senescence index (**d**) as in (**a**). The cotyledon and unifoliate leaf senescence index was calculated at the age of 15 days and 25 days, respectively ($n \geq 20$ biological replicates). **e** Relative transcript levels of senescence marker genes *GmSAG12* in the unifoliate leaves as in (**a**). Values are means ± SD (n = 3 biological replicates). P values were calculated by unpaired two-tailed t-test. Source data are provided as a Source Data file.

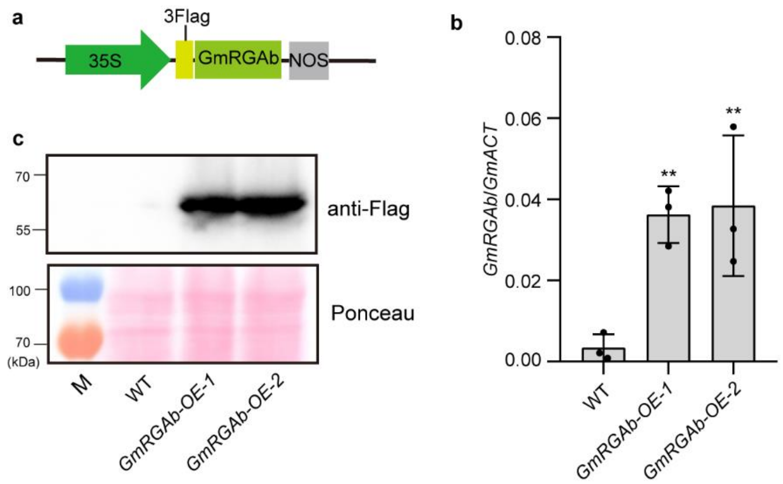

Supplementary Fig. 22: Molecular identification of GmRGAb overexpression in soybean. **a** Construct scheme of GmRGAb overexpression, with Flag tag. **b** Transcriptional levels of *GmRGAb* in wild type and *GmRGAb* overexpressing lines under long-day conditions at 10 days after planting. Values are means ± SD (n = 3 biological replicates). P values were calculated by unpaired two-tailed t-test. $^{**}p < 0.01$. **c** Immunoblots show the presence of 3×Flag-GmRGAb fusion protein in the transgenic plants using anti-Flag antibody. M, protein marker. Source data are provided as a Source Data file.

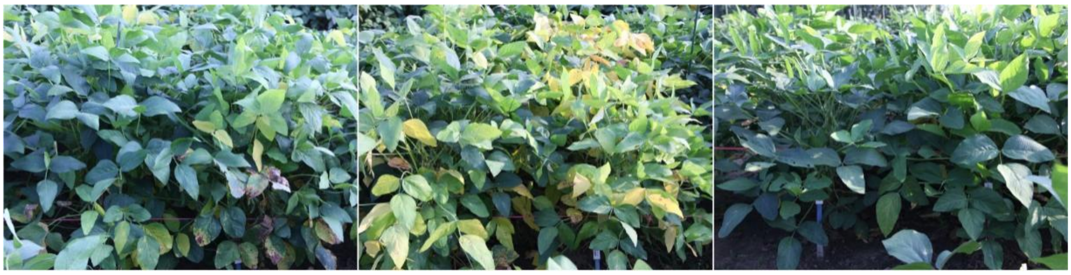

Supplementary Fig. 23: Leaf senescence performance of DELLA proteins mutants and wild-type plants under natural field conditions. Representative images of wild-type, *Gmrgas-dm* mutant, and *GmRGAb*-OE-1 plants grown under natural field conditions at the age of 97 days after sowing.

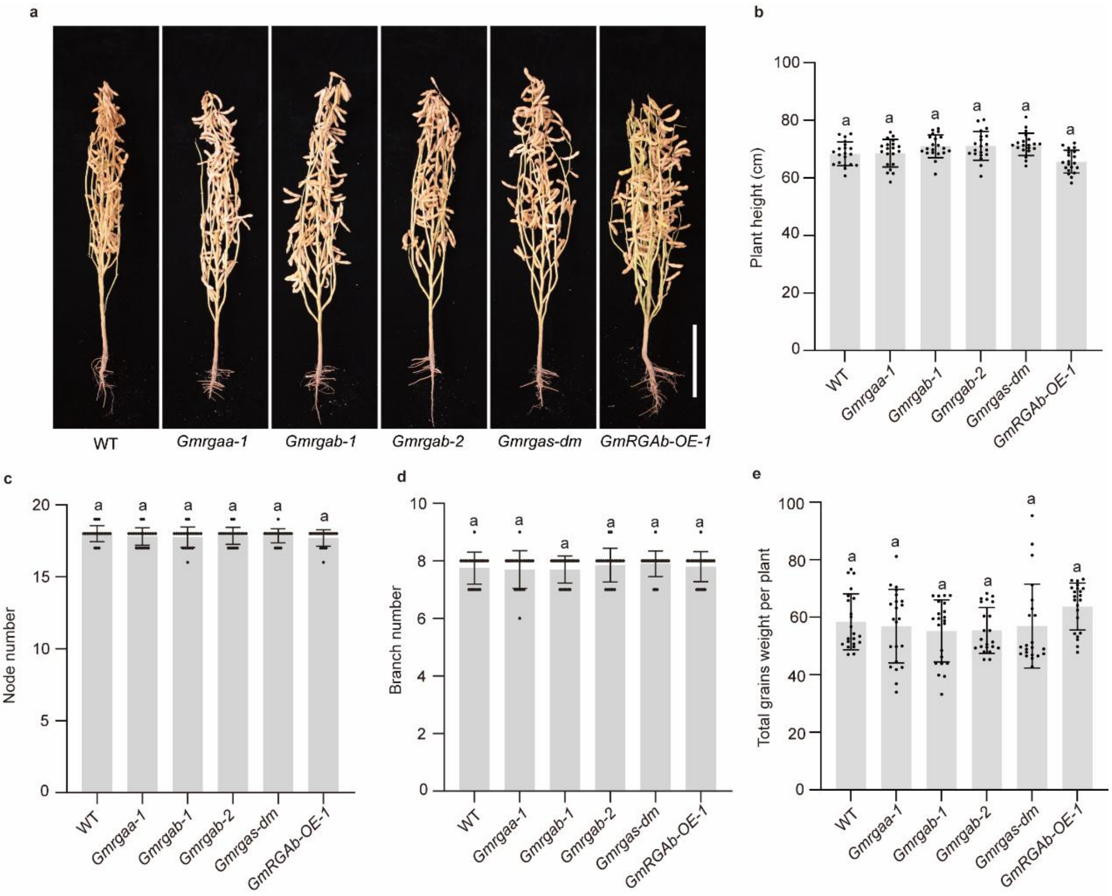

Supplementary Fig. 24: Phenotypic and yield trait characteristics of *Gmrga* mutants and *GmRGAb* overexpression line under natural field conditions. a Photographs of the indicated lines at the R8 stage are shown. Scale bar, 20 cm. **b-e** Phenotypic comparison among *Gmrgas* mutants and *GmRGAb* overexpression line in plant height (**b**), node number (**c**), branch number (**d**), and grain weight per plant (**e**). Values are means ± SD (n = 30 biologically independent plants). The lowercase letters indicate significant differences ($P < 0.05$, ANOVA with Tukey's post hoc test). Source data are provided as a Source Data file.

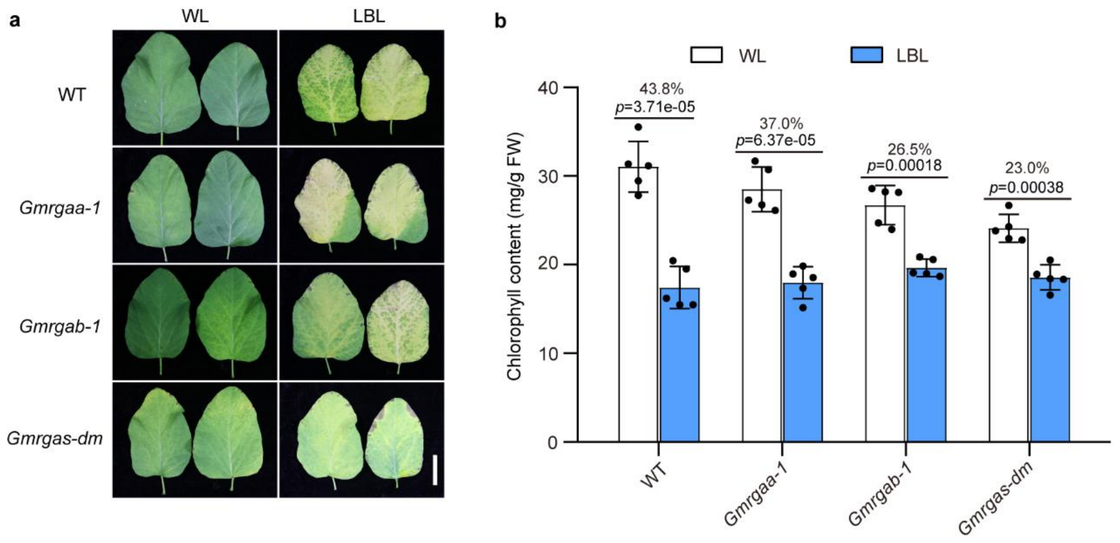

Supplementary Fig. 25: DELLA proteins are essential for LBL-induced leaf senescence in soybean. a Leaf senescence phenotype of *Gmrgaa-1*, *Gmrgab-1*, *Gmrgas-dm* mutant, and wild-type plants under WL or LBL conditions. Seedlings were grown under continuous white light for 10 days, then a pair of unifoliate leaves were treated with different light regimes (LBL or WL) for 15 days. Scale bar, 3 cm. **b** Chlorophyll content in the unifoliate leaves as in (**a**). Values are means ± SD (n = 5 biologically independent plants). P values were calculated by unpaired two-tailed t-test. The percentage decrease in chlorophyll content under WL compared to LBL is indicated by the values above the respective p values. Source data are provided as a Source Data file.

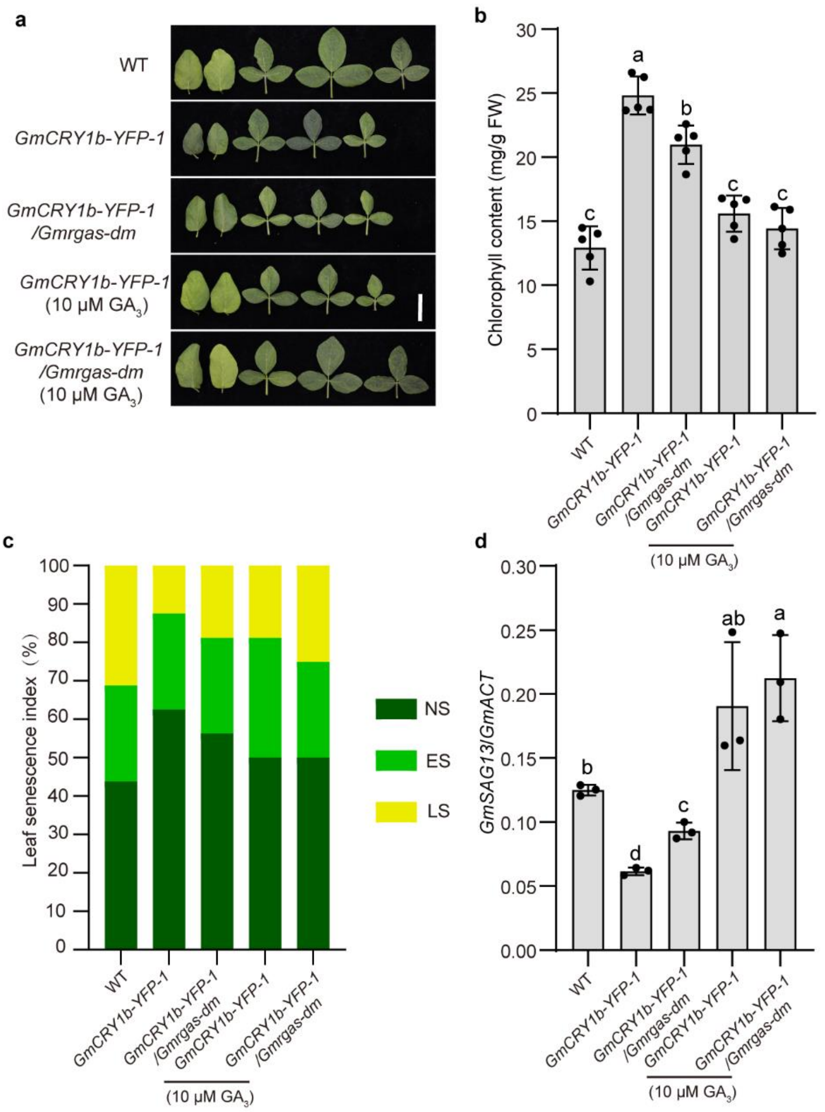

Supplementary Fig. 26: Influence of DELLA protein mutants and GA application on suppression of GmCRY1s-mediated delayed leaf senescence in soybean. **a** Comparison of leaf senescence phenotypes between *GmCRY1b-YFP-1*, *GmCRY1b-YFP-1/Gmrgas-dm*, wild-type plants cultivated for 26 days under long-day conditions. For GA_3 treatment, 6-day-old seedlings of *GmCRY1b-YFP-1* and *GmCRY1b-YFP-1/Gmrgas-dm* lines were with 10 μM GA_3 for 20 days respectively. Scale bar, 5 cm. **b** Chlorophyll content of the indicated lines as in (**a**). Values are means ± SD (n = 5 biologically independent plants). **c** Leaf senescence index of the indicated lines as in (**a**). **d** Relative expression levels of senescence marker gene *GmSAG13* in the unifoliate leaf of 20-day-old seedling. Values are mean ± SD (n = 3 biological replicates). Statistical significant differences were determined by ANOVA with Tukey's post hoc test (lowercase letters indicate $P < 0.05$). Source data are provided as a Source Data file.

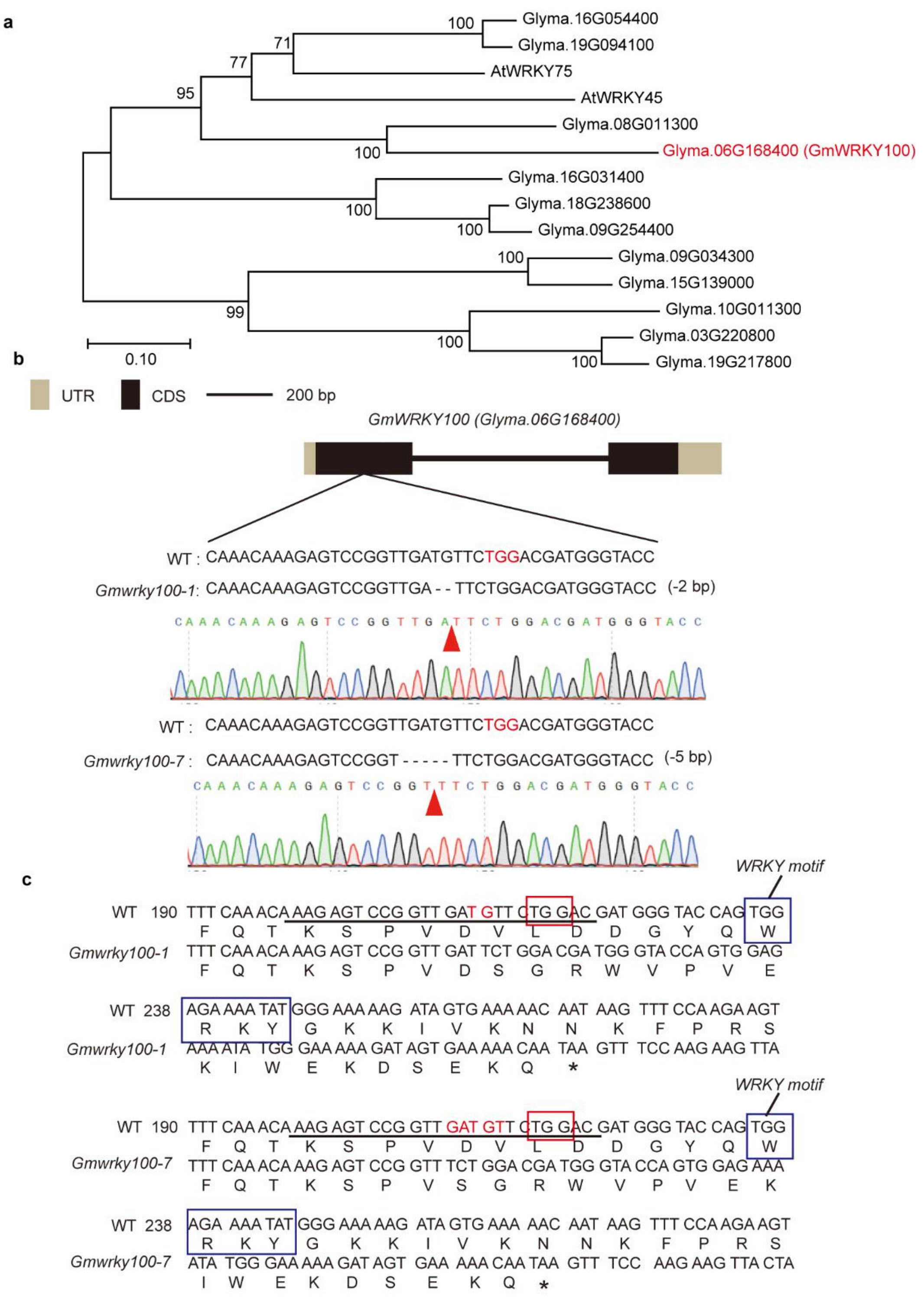

Supplementary Fig. 27: Phylogenetic tree of GmWRKY100 proteins in *Arabidopsis* and *Glycine max,* and generation of *Gmwrky100* mutants by CRISPR/Cas9 system in soybean. a A phylogenetic tree based on the amino acid sequences of GmWRKY100 proteins and their homologous proteins in *Arabidopsis* and *Glycine max*. The tree was constructed using the neighbor-joining method. Database for searching: Phytozome (http://phytozome-next.jgj.doe.gov), TAIR10

(http://arabidopsis.org/index.jsp). **b** CRISPR/Cas9-engineered mutations in *GmWRKY100* gene. CRISPR/Cas9-induced 2 bp deletion and 5 bp deletion in the first exon of *GmWRKY100* detected by sanger sequencing, respectively. Red arrowheads represent the cleavage site. **c** Sequence comparison of wild-type TL1 cultivar and *GmWRKY100* mutants. The underlined nucleotides indicate the target sites; The red boxes indicate the protospacer-adjacent motif (PAM); The blue boxes indicate the conserved amino acid sequence in the WRKY transcription factor. An asterisk indicates the termination of translation.

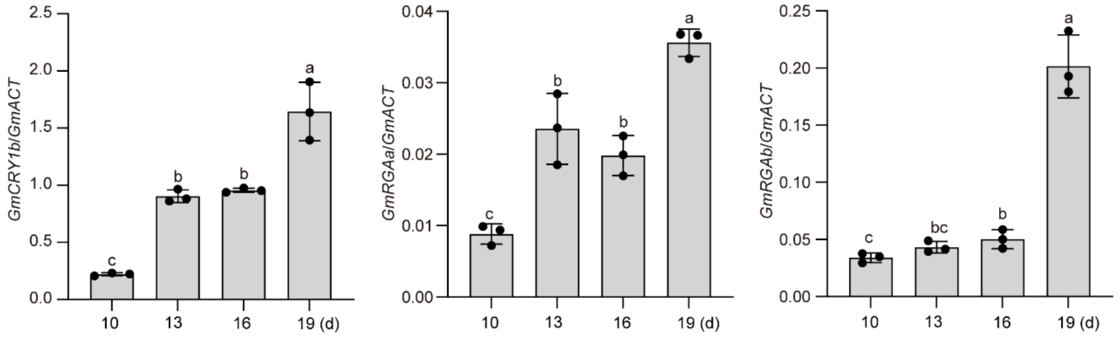

Supplementary Fig. 28: Relative transcript levels of *GmCRY1b, GmRGAa* and *GmRGAb* in gradually aged leaves. The unifoliate leaves of wild-type TL1 cultivar were collected at the indicated leaf age under long-day conditions. Values are means ± SD (n = 3 biological replicates). Different lowercase letters indicate significant differences ($P < 0.05$, ANOVA with Tukey's post hoc test). Source data are provided as a Source Data file.

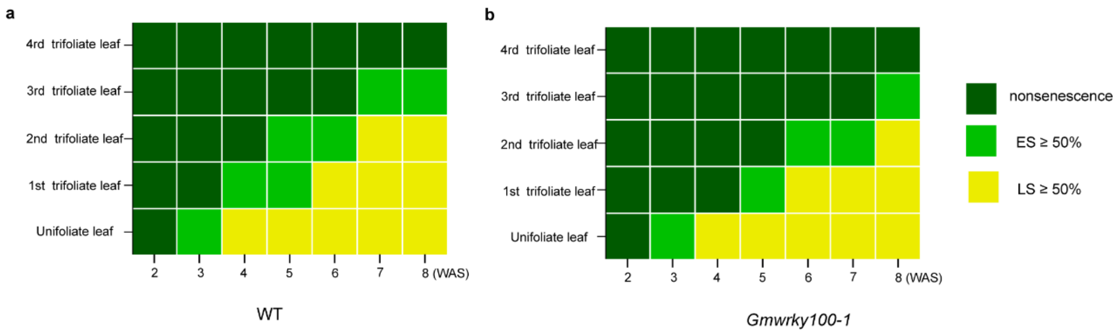

Supplementary Fig. 29: Leaf senescence of the wild-type plants and *Gmwrky100* mutant under natural field conditions. Senescence trend of unifoliate leaves and main stem trifoliate leaves in the wild-type plant (**a**), and *Gmwrky100-1* mutant (**b**) at indicated weeks after sowing (WAS). NS, fully expanded mature cotyledons without senescence symptoms; ES, early senescent stage with <25% cotyledons or leaf area yellowing; LS, late senescent stage with over 60% cotyledons or leaf area yellowing (*n* = 10 biologically independent plants). Source data are provided as a Source Data file.

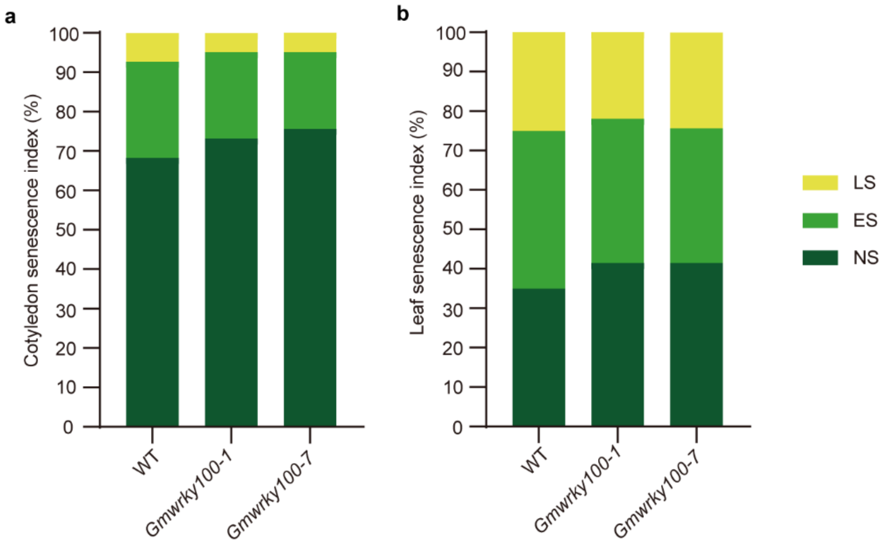

Supplementary Fig. 30: Leaf senescence analysis of *Gmwrky100* mutants and wild-type plants in soybean. Cotyledon senescence index (**a**) and unifoliate leaves senescence index (**b**) of *Gmwrky100* mutants and wild-type plants. The cotyledon and unifoliate leaves senescence indexes were calculated at the age of 15 days and 25 days respectively ($n \geq 20$ biological replicates). Source data are provided as a Source Data file.

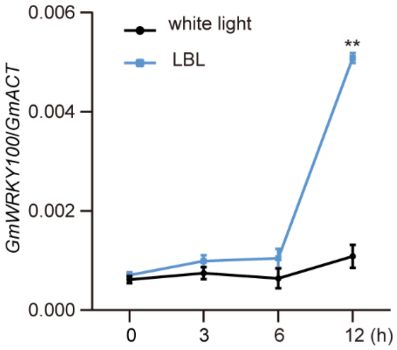

Supplementary Fig. 31: Low blue light induces the expression of *GmWRKY100*. Quantitative RT-PCR analysis of *GmWRKY100* expression in the wild-type TL1 cultivar. 10-day-old etiolated Seedlings were transformed to white light or LBL conditions for the indicated time. Values are means ± SD ($n = 3$ biological replicates), $^{**}P < 0.01$ by unpaired two-tailed t-test. Source data are provided as a Source Data file.

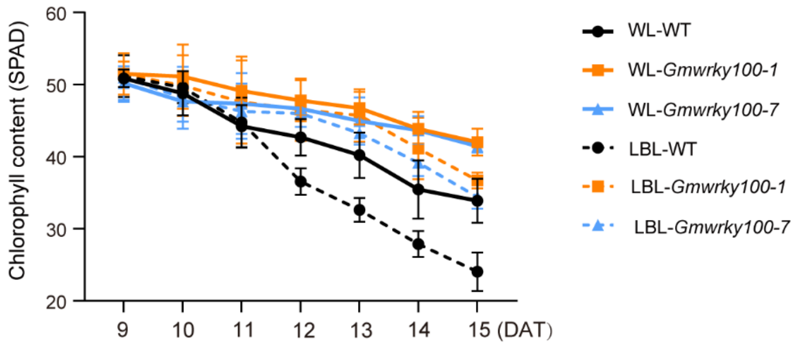

Supplementary Fig. 32: Chlorophyll content dynamics in the unifoliate leaves of indicated lines under white light and LBL conditions. Seedlings cultivated under long-day conditions for 10 days had their unifoliate leaves exposed either to LBL or white light, as depicted in Figure 1a. Daily measurements of chlorophyll content, as represented by SPAD, were taken 1 hour post illumination using a chlorophyll meter SPAD-502. Values are means ± SD ($n \geq 5$ biological replicates). DAT indicates the days after LBL treatment. Source data are provided as a Source Data file.

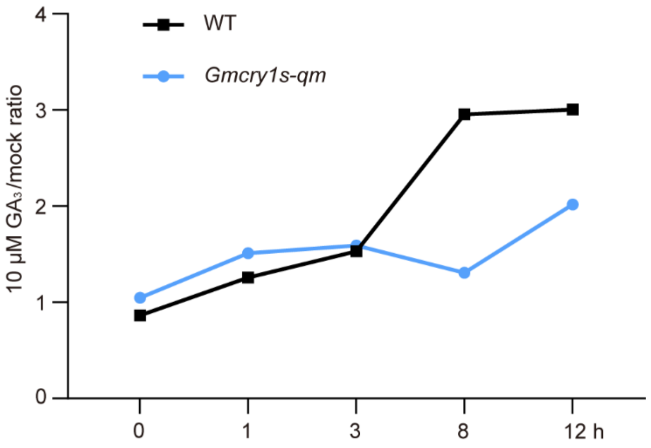

Supplementary Fig. 33: Comparison of GA-triggered expression of *GmWRKY100* in wild-type plant and *Gmcry1-qm* mutant. Seedlings were grown under continuous WL conditions for 14 days, then exogenously treated with 10 μM GA_3. The unifoliate leaves were collected for RT-qPCR analysis at the indicated time after treatment. Source data are provided as a Source Data file.

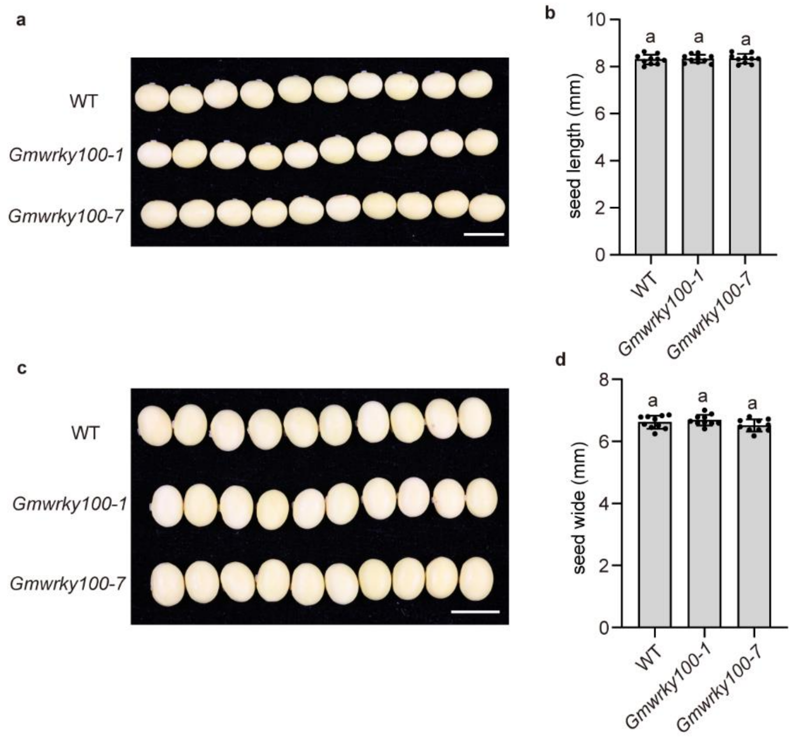

Supplementary Fig. 34: Comparison of mature seeds of WT, *Gmwrky100-1* and *Gmwrky100-7* plants. a and c Seed image of indicated lines. Scale bar, 1 cm. **b and d** Quantitative analysis of seed length (**b**) and seed width (**d**) of WT, *Gmwrky100-1* and *Gmwrky100-7* plants. Values are means ± SD (n = 10 biological replicates). Lowercase letters indicate significant differences ($P < 0.05$, ANOVA with Tukey's post hoc test). Source data are provided as a Source Data file.

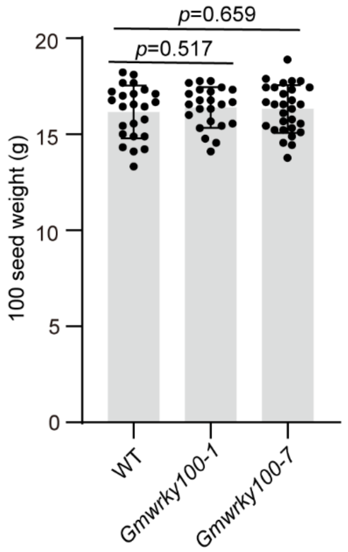

Supplementary Fig. 35: Comparison of 100 seed weight of WT, *Gmwrky100-1* and *Gmwrky100-7* plants. Values are means ± SD (n = 30 biologically independent plants). P values were calculated by unpaired two-tailed t-test. Source data are provided as a Source Data file.

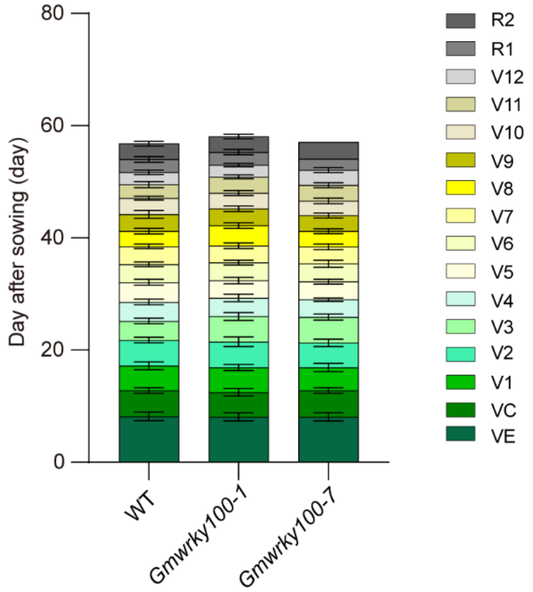

Supplementary Fig. 36: Developmental stage comparation between wild-type plants and *Gmwrky100* mutants under natural field conditions. Values are means ± SD (n = 10 biologically independent plants). Source data are provided as a Source Data file.
