# Supplementary material for: The mechanism of low blue light-induced leaf senescence mediated by GmCRY1s in soybean
Source: Nat Commun. 2024 Jan 27;15:798. doi: 10.1038/s41467-024-45086-5 (PMC10821915; doi:10.1038/s41467-024-45086-5)
Supplement: Supplementary file 3 — Description of Additional Supplementary Files [file 41467_2024_45086_MOESM3_ESM.pdf]

Description of Additional Supplementary Files:

Supplementary Data 1: Primers used in this study.

Supplementary Data 2: Putative GmCRY1b-interacting proteins indentified by Y2H

Supplementary Data 3: Candidate differential expression genes that regulate leaf senescence
