# Supplementary material for: The mechanism of low blue light-induced leaf senescence mediated by GmCRY1s in soybean
Source: Nat Commun. 2024 Jan 27;15:798. doi: 10.1038/s41467-024-45086-5 (PMC10821915; doi:10.1038/s41467-024-45086-5)
Supplement: Supplementary file 7 — Reporting Summary [file 41467_2024_45086_MOESM7_ESM.pdf]

nature portfolio

Corresponding author(s): Bin Liu

Last updated by author(s): 2024/1/12

# Reporting Summary

Nature Portfolio wishes to improve the reproducibility of the work that we publish. This form provides structure for consistency and transparency in reporting. For further information on Nature Portfolio policies, see our Editorial Policies and the Editorial Policy Checklist.

## Statistics

For all statistical analyses, confirm that the following items are present in the figure legend, table legend, main text, or Methods section.

| n/a | Confirmed | |
|---|---|---|
| ☐ | ☒ | The exact sample size (*n*) for each experimental group/condition, given as a discrete number and unit of measurement |
| ☐ | ☒ | A statement on whether measurements were taken from distinct samples or whether the same sample was measured repeatedly |
| ☐ | ☒ | The statistical test(s) used AND whether they are one- or two-sided<br>*Only common tests should be described solely by name; describe more complex techniques in the Methods section.* |
| ☒ | ☐ | A description of all covariates tested |
| ☒ | ☐ | A description of any assumptions or corrections, such as tests of normality and adjustment for multiple comparisons |
| ☐ | ☒ | A full description of the statistical parameters including central tendency (e.g. means) or other basic estimates (e.g. regression coefficient) AND variation (e.g. standard deviation) or associated estimates of uncertainty (e.g. confidence intervals) |
| ☐ | ☒ | For null hypothesis testing, the test statistic (e.g. *F*, *t*, *r*) with confidence intervals, effect sizes, degrees of freedom and *P* value noted<br>*Give P values as exact values whenever suitable.* |
| ☒ | ☐ | For Bayesian analysis, information on the choice of priors and Markov chain Monte Carlo settings |
| ☒ | ☐ | For hierarchical and complex designs, identification of the appropriate level for tests and full reporting of outcomes |
| ☒ | ☐ | Estimates of effect sizes (e.g. Cohen's *d*, Pearson's *r*), indicating how they were calculated |

*Our web collection on statistics for biologists contains articles on many of the points above.*

## Software and code

Policy information about availability of computer code

| | |
|---|---|
| Data collection | Confocal Microscopy: Zeiss LSM980<br>LUC and REN activities analysis: Centro XS3 LB 960<br>Photogragh: Canon EOS 5D Mark II<br>Western Blot: Tanon-5200<br>RT-qPCR: qTOWER 3G |
| Data analysis | Quantitation of protein abundance: ImageJ V2.3.0<br>Data analysis: Graphpad Prism V9.4.1, Microsoft Excel<br>Phylogenetic tree: MEGA V10.2.6 |

For manuscripts utilizing custom algorithms or software that are central to the research but not yet described in published literature, software must be made available to editors and reviewers. We strongly encourage code deposition in a community repository (e.g. GitHub). See the Nature Portfolio guidelines for submitting code & software for further information.

# Data

Policy information about availability of data

All manuscripts must include a data availability statement. This statement should provide the following information, where applicable:

- Accession codes, unique identifiers, or web links for publicly available datasets
- A description of any restrictions on data availability
- For clinical datasets or third party data, please ensure that the statement adheres to our policy

All the data generated in this study are provided in the Supplementary Information and Source Data file. Source data are provided with this paper.

# Research involving human participants, their data, or biological material

Policy information about studies with human participants or human data. See also policy information about sex, gender (identity/presentation), and sexual orientation and race, ethnicity and racism.

| | |
|---|---|
| Reporting on sex and gender | not applicable |
| Reporting on race, ethnicity, or other socially relevant groupings | not applicable |
| Population characteristics | not applicable |
| Recruitment | not applicable |
| Ethics oversight | not applicable |

Note that full information on the approval of the study protocol must also be provided in the manuscript.

# Field-specific reporting

Please select the one below that is the best fit for your research. If you are not sure, read the appropriate sections before making your selection.

- [x] Life sciences
- [ ] Behavioural & social sciences
- [ ] Ecological, evolutionary & environmental sciences

For a reference copy of the document with all sections, see nature.com/documents/nr-reporting-summary-flat.pdf

# Life sciences study design

All studies must disclose on these points even when the disclosure is negative.

| | |
|---|---|
| Sample size | Sample sizes were determined based on preliminary data demonstrating statistically significant differences for each specific assay. |
| Data exclusions | No data were excluded from the analysis. |
| Replication | All experiments were performed with at least three technical replicates on more than one occasion to ensure reproducibility across experiments. In each case, one of the them was presented in the figures. |
| Randomization | Soybean plants grown in the plant growth chambers or natural field conditions were randomized along with all required controls. The samples tested were randomized and replicated in all experiments. |
| Blinding | Blinding was not relevant as all processing methods were done through available software with consistent parameters utilized across all soybean plants and required controls. |

# Reporting for specific materials, systems and methods

We require information from authors about some types of materials, experimental systems and methods used in many studies. Here, indicate whether each material, system or method listed is relevant to your study. If you are not sure if a list item applies to your research, read the appropriate section before selecting a response.

## Materials & experimental systems

| n/a | Involved in the study | |
|---|---|---|
| ☐ | ☒ | Antibodies |
| ☒ | ☐ | Eukaryotic cell lines |
| ☒ | ☐ | Palaeontology and archaeology |
| ☒ | ☐ | Animals and other organisms |
| ☒ | ☐ | Clinical data |
| ☒ | ☐ | Dual use research of concern |
| ☐ | ☒ | Plants |

## Methods

| n/a | Involved in the study | |
|---|---|---|
| ☒ | ☐ | ChIP-seq |
| ☒ | ☐ | Flow cytometry |
| ☒ | ☐ | MRI-based neuroimaging |

# Antibodies

Antibodies used

Commercial antibodies: anti-GFP (MBL, 598, 1:2000 dilution), anti-Flag (Abmart, M20008L, 1:5000 dilution), Goat anti-Rabit lgG-HRP (Abmart, M21002, 1:2000 dilution), anti-HSP82 (BPI, AbM51099-31-PU, 1:10000 dilution)

Validation

anti-GFP (https://ruo.mbl.co.jp/bio/e/sch/A/?kw=598&kwdrop=on&pcd=&cln=&sig=&iso=&pnum=100&od=6&aps=1),anti-Flag (http://www.ab-mart.com.cn/product.aspx?id=3&f=cn&keys=M20008L), Goat anti-Rabit lgG-HRP (http://www.ab-mart.com.cn/product.aspx?id=3&f=cn&keys=M21002), anti-HSP82 (http://www.proteomics.org.cn/product/202.html)

# Dual use research of concern

Policy information about dual use research of concern

## Hazards

Could the accidental, deliberate or reckless misuse of agents or technologies generated in the work, or the application of information presented in the manuscript, pose a threat to:

| No | Yes | |
|---|---|---|
| ☒ | ☐ | Public health |
| ☒ | ☐ | National security |
| ☒ | ☐ | Crops and/or livestock |
| ☒ | ☐ | Ecosystems |
| ☒ | ☐ | Any other significant area |

## Experiments of concern

Does the work involve any of these experiments of concern:

| No | Yes | |
|---|---|---|
| ☒ | ☐ | Demonstrate how to render a vaccine ineffective |
| ☒ | ☐ | Confer resistance to therapeutically useful antibiotics or antiviral agents |
| ☒ | ☐ | Enhance the virulence of a pathogen or render a nonpathogen virulent |
| ☒ | ☐ | Increase transmissibility of a pathogen |
| ☒ | ☐ | Alter the host range of a pathogen |
| ☒ | ☐ | Enable evasion of diagnostic/detection modalities |
| ☒ | ☐ | Enable the weaponization of a biological agent or toxin |
| ☒ | ☐ | Any other potentially harmful combination of experiments and agents |

## Plants

| | |
|---|---|
| Seed stocks | The elite soybean cultivar Tian-Long 1 was used as the WT control in this study, which was provided by the Oil Crops Research Institute of the Chinese Academy of Agricultural Sciences. Accession number:s2228 |
| Novel plant genotypes | The present study does not involve novel plant genotypes. |
| Authentication | N/A |
